# Rapamycin limits CD4^+^ T cell proliferation in simian immunodeficiency virus–infected rhesus macaques on antiretroviral therapy

**DOI:** 10.1172/JCI156063

**Published:** 2022-05-16

**Authors:** Benjamin D. Varco-Merth, William Brantley, Alejandra Marenco, Derick D. Duell, Devin N. Fachko, Brian Richardson, Kathleen Busman-Sahay, Danica Shao, Walter Flores, Kathleen Engelman, Yoshinori Fukazawa, Scott W. Wong, Rebecca L. Skalsky, Jeremy Smedley, Michael K. Axthelm, Jeffrey D. Lifson, Jacob D. Estes, Paul T. Edlefsen, Louis J. Picker, Cheryl M.A. Cameron, Timothy J. Henrich, Afam A. Okoye

**Affiliations:** 1 Vaccine and Gene Therapy Institute and; 2 Oregon National Primate Research Center, Oregon Health & Science University, Beaverton, Oregon, USA.; 3 Department of Nutrition, School of Medicine, Case Western Reserve University, Cleveland Ohio, USA.; 4 Statistical Center for HIV/AIDS Research and Prevention, Vaccine and Infectious Disease Division, Fred Hutchinson Cancer Research Center, Seattle, Washington, USA.; 5 MassBiologics, University of Massachusetts Medical School, Boston, Massachusetts, USA.; 6 AIDS and Cancer Virus Program, Leidos Biomedical Research Inc., Frederick National Laboratory, Frederick, Maryland, USA.; 7 Department of Medicine, UCSF, San Francisco, California, USA.

**Keywords:** AIDS/HIV, Homeostasis, T cells

## Abstract

Proliferation of latently infected CD4^+^ T cells with replication-competent proviruses is an important mechanism contributing to HIV persistence during antiretroviral therapy (ART). One approach to targeting this latent cell expansion is to inhibit mTOR, a regulatory kinase involved with cell growth, metabolism, and proliferation. Here, we determined the effects of chronic mTOR inhibition with rapamycin with or without T cell activation in SIV-infected rhesus macaques (RMs) on ART. Rapamycin perturbed the expression of multiple genes and signaling pathways important for cellular proliferation and substantially decreased the frequency of proliferating CD4^+^ memory T cells (TM cells) in blood and tissues. However, levels of cell-associated SIV DNA and SIV RNA were not markedly different between rapamycin-treated RMs and controls during ART. T cell activation with an anti-CD3LALA antibody induced increases in SIV RNA in plasma of RMs on rapamycin, consistent with SIV production. However, upon ART cessation, both rapamycin and CD3LALA–treated and control-treated RMs rebounded in less than 12 days, with no difference in the time to viral rebound or post-ART viral load set points. These results indicate that, while rapamycin can decrease the proliferation of CD4^+^ TM cells, chronic mTOR inhibition alone or in combination with T cell activation was not sufficient to disrupt the stability of the SIV reservoir.

## Introduction

The advent of antiretroviral therapy (ART) has dramatically improved the life expectancy of people with HIV (PWH) infection. However, current ART regimens act by blocking de novo infection of uninfected cells and do not affect already infected cells. Thus, ART alone is not a cure and PWH are required to remain on treatment for life to prevent the resumption of viral replication and disease progression. Even during sustained ART that effectively suppresses viral replication, a pool of CD4^+^ T cells harboring integrated, replication-competent HIV proviruses persists and can give rise to recrudescent spreading infection if ART is discontinued (1–3). These latently infected CD4^+^ T cells can persist for long periods of time and are believed to be maintained by mechanisms that allow them to proliferate and expand during ART (4–6). Indeed, homeostatic proliferation of CD4^+^ transitional memory T cells (TrM cells) has been described as a major factor contributing to the stability of the HIV reservoir during ART (7). Additionally, HIV integration site analyses have shown HIV proviruses can integrate in genomic regions encoding potential oncogenes as well as genes that are associated with cell-cycle progression (8–11). However, HIV integration in oncogenes may only play a minor role in reservoir persistence (12). Large proportions of unique HIV integrations are observed in clonally expanded cells, suggesting preferential survival and/or proliferative expansion of select clonal populations during ART (9, 10). This expansion of clonally selected cells can often be driven by the proliferation of antigen-specific T cells exposed to persistent antigens (13, 14). However, cytokine-driven proliferation may also play an important role (15). Crucially, proliferation of latently infected CD4^+^ T cells can occur without HIV proviral gene expression, which would prevent immune recognition and clearance (16). Therefore, restricting proliferation of CD4^+^ T cells harboring intact, replication-competent HIV proviruses has been proposed as an effective strategy for disrupting the long-term stability of the HIV reservoir in PWH. Indeed, this concept is supported by mathematical modeling that suggests antiproliferative therapies in PWH could accelerate the decay of the HIV reservoir in contrast with ART alone (17). Antiproliferative agents may also act in synergy with latency-reversing agents (LRAs) to limit CD4^+^ T cell proliferation and potential expansion of the pool of persistently infected cells following activation, addressing an important concern regarding the use of LRAs (18).

One approach to limiting the proliferative capacity of CD4^+^ T cells is to inhibit the activity of mTOR, a highly conserved serine-threonine protein kinase that plays a central role in regulating cell growth, cell-cycle progression, and lipid and glucose metabolism (19). mTOR signaling can be activated by a variety of stimuli, including growth factors, cytokines, amino acids, oxygen, energy, and even infectious agents. mTOR exists in 2 distinct protein complexes, mTOR complex 1 (mTORC1) and mTORC2, which differ in their regulation and downstream targets (19). Rapamycin (sirolimus) is a specific inhibitor of mTOR via direct interactions with mTORC1. In contrast, mTORC2 is relatively resistant to rapamycin, but its activity is reduced at higher doses or following prolonged exposure (20, 21). Inhibition of mTOR results in reduced T cell proliferation, differentiation, and activation. Therefore, mTOR inhibitors such as rapamycin and its analogues, often referred to as rapalogs, are commonly used as antiproliferative agents in cancer therapies or for solid-organ transplantation to prevent graft rejection (22–25).

mTOR activity has also been implicated as a multifactorial regulator of HIV infection. HIV upregulates mTOR signaling following infection, which increases the pool of metabolites required to support virus replication (26–28). The susceptibility of CD4^+^ T cells to HIV infection has also been linked with mTOR signaling, as inhibition of mTOR with rapamycin decreases expression of the HIV coreceptor CCR5 on T cells (29, 30), and mTOR has also been implicated in regulating HIV latency, with inhibition of mTOR shown to suppress HIV reactivation (31, 32). These observations have led to suggestions that mTOR inhibitors could be effective in so-called “block and lock” HIV cure strategies that aim to induce a state of deep HIV latency, i.e., durable suppression of HIV gene transcription in the absence of ART (33, 34). Interestingly, reduced HIV burden has been observed in PWH who received mTOR inhibitors as part of an immunosuppressive protocol for solid organ transplantation (35–38).

The goals of this study were 2-fold: (a) to validate the use of rapamycin in SIV-infected rhesus macaques (RMs) on ART, documenting changes in metabolism, gene expression, and immune cell homeostasis in comparison with humans and rodent models for which these effects have been well characterized; and (b) to characterize the effects of chronic mTOR inhibition with rapamycin on CD4^+^ T cell proliferation in blood and tissues, SIV reservoir dynamics during ART, and rebound kinetics following ART release. We also assessed whether rapamycin could be combined with a potent activating T cell receptor agonist to safely induce SIV gene expression and reduce the frequency of latently infected cells while also avoiding proliferative expansion of the persistent virus pool and minimizing toxicity. Our analyses revealed that chronic mTOR inhibition with rapamycin had profound effects on CD4^+^ T cell homeostasis, including substantial changes in chemokine receptor expression and reduced frequencies of proliferating CD4^+^ T cells in blood and tissues. Administration of an anti-CD3LALA mAb in RMs on rapamycin was safe and resulted in increased T cell activation and plasma viremia, consistent with the induction of SIV production. However, rapamycin in combination with anti-CD3LALA had no durable effect on cell-associated viral loads during ART or on viral rebound kinetics following ART withdrawal.

## Results

### Study design.

**Figure 1:**
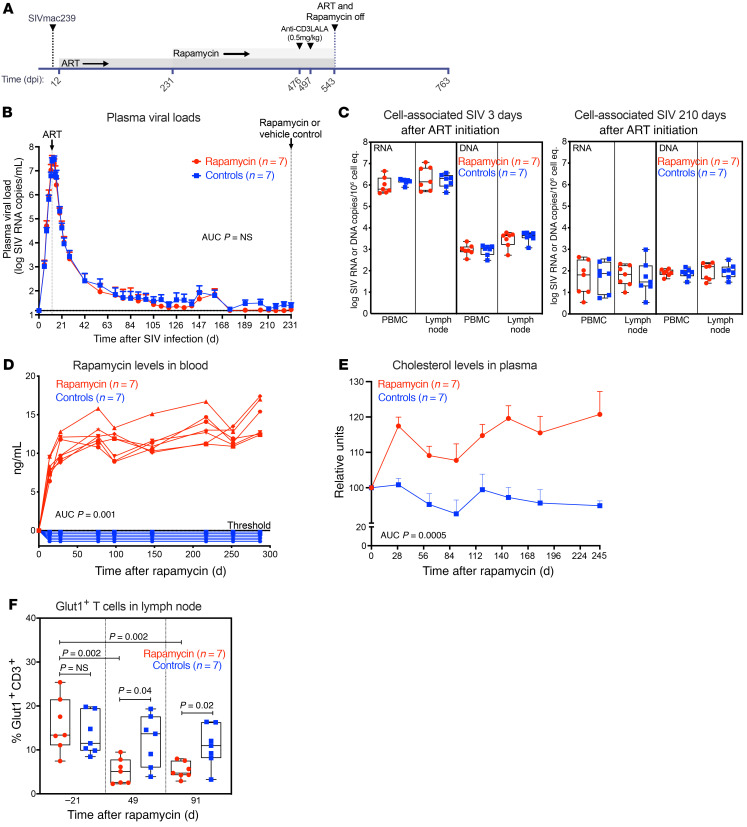
Plasma and cell-associated viral loads were equivalent between study groups prior to rapamycin treatment. (**A**) Schematic representation of the study protocol showing SIVmac239M infection, ART initiation 12 days dpi, rapamycin or vehicle control administration, which occurred daily from 231 to 543 dpi, and anti-CD3LALA infusion in rapamycin-treated RMs on 467 and 497 dpi. (**B**) Mean (+SEM) pvl profiles of rapamycin (red) or vehicle controls (blue) (*n* = 7 each) prior to treatment initiation. (**C**) Comparison of SIV RNA and DNA levels in PBMCs and LNs (copies per 10^6^ cell equivalents) between rapamycin (red) and vehicle controls (blue) at 3 days (15 dpi) and 210 days after ART. (**D**) Quantification of rapamycin drug levels in plasma. (**E**) Mean (+SEM) change from baseline cholesterol levels in plasma of rapamycin-treated RMs (red) and vehicle controls. (**F**) Quantification of the number of Glut1^+^ T cells per 10^5^ cells in LNs at –21, 49, and 91 days after initiation of rapamycin or vehicle in the treatment groups. Each data point represents the average number of Glut1^+^CD3^+^ T cells derived from quantitative measures from 2 to 3 LN sections from a single time point from an individual RM. The WRS test was used to determine the significance of differences between the rapamycin or vehicle control treatment groups (*P* values ≤ 0.05 are shown). Box plots show jittered points, a box from 1st to 3rd quartiles (IQR), and a line at the median, with whiskers extending to the farthest data point within 1.5× IQR above and below the box.

The experimental protocol included 14 adult male RMs (Supplemental Table 1; supplemental material available online with this article; https://doi.org/10.1172/JCI156063DS1) that were intravenously inoculated with 200 infectious units (IU) of SIVmac239 before starting ART 12 days post infection (dpi). We started ART 12 dpi based on previous observations showing that ART initiation at time of peak plasma viremia (i.e., 12 dpi) allows for maximal or near-maximal seeding of a rebound-competent SIV reservoir while also allowing for virus suppression within an experimentally feasible time frame (39, 40). At 231 dpi, RMs were divided into 2 treatment groups of 7 RMs each that received twice daily intramuscular injections of rapamycin or vehicle control for up to 44 weeks (Figure 1A). As shown in Figure 1B, mean (+SEM) plasma viral loads (pvl) were statistically indistinguishable between both groups prior to rapamycin treatment. In addition, levels of cell-associated SIV DNA and SIV RNA were equivalent between treatment groups at 3 days after ART initiation (15 dpi) and at 3 weeks prior to the start of rapamycin treatment (210 dpi) (Figure 1C).

### Drug levels, pharmacodynamics, and safety monitoring.

After 4 weeks of twice daily rapamycin administration, we achieved trough drug levels greater than 8 ng/ml, which were maintained throughout the treatment period (Figure 1D). We monitored RMs using a comprehensive metabolic panel to assess for any toxicities and to document pharmacodynamic activity of the administered rapamycin, and we observed a significant increase in levels of total cholesterol in rapamycin-treated RMs (Figure 1E). This increase is consistent with previous reports of disrupted lipid metabolism following mTOR inhibition (41, 42). While most other analytes, including blood glucose, were unaffected by rapamycin, triglyceride levels were slightly elevated. In contrast, both potassium and blood urea nitrogen levels were decreased in rapamycin-treated RMs relative to controls (Supplemental Figure 1A). In general, this suggests rapamycin dosing in SIV-infected RMs on ART can induce considerable metabolic impairments. We also assessed plasma levels of d-dimer, a marker of coagulation (43), as well as soluble CD14 (sCD14) and LPS, both of which have been used as surrogate markers of microbial translocation (44). We found increased d-dimer levels in the plasma of rapamycin-treated RMs relative to controls (Supplemental Figure 1B), possibly associated with the role of mTOR in platelets (45, 46). In contrast, both sCD14 and LPS levels were similar between treatment groups over time (Supplemental Figure 1B).

We also assessed changes in metabolic hormones in plasma at 34 weeks after rapamycin treatment, coinciding with the time of increased total cholesterol, and observed a substantial increase in the gastric hormone ghrelin in rapamycin-treated RMs (Supplemental Figure 2). This result supports previous observations showing ghrelin production is regulated by mTOR signaling (47). The gut hormone peptide YY, which is produced by gastrointestinal L cells, was also elevated in rapamycin-treated RMs. Other metabolic markers, including C-peptide, glucagon, amylin, pancreatic polypeptide, glucagon-like peptide-1, and insulin, were not significantly different between rapamycin-treated RMs and controls, although an increase in levels of the gastric inhibitory polypeptide was observed. There was also an increase in levels of monocyte chemoattractant protein-1 (MCP-1) in rapamycin-treated RMs. MCP-1 is a proinflammatory cytokine that regulates the migration and infiltration of monocytes and macrophages, and studies in mice and humans have shown rapamycin treatment can increase MCP-1 mRNA expression (48, 49). To further characterize the effects of rapamycin, we screened lymph node (LN) sections for expression of glucose transporter type 1 (Glut1) on T cells. Glut1 is a protein that facilitates glucose transport across the cell membrane, and its expression is directly regulated by mTOR signaling (50). As shown in Figure 1F, the frequencies of Glut1^+^ T cells present in the LNs were reduced in rapamycin-treated RMs relative to controls, indicating substantial perturbations in mTOR signaling in lymphoid tissues.

### Effects of rapamycin on circulating miRNAs.

**Figure 2:**
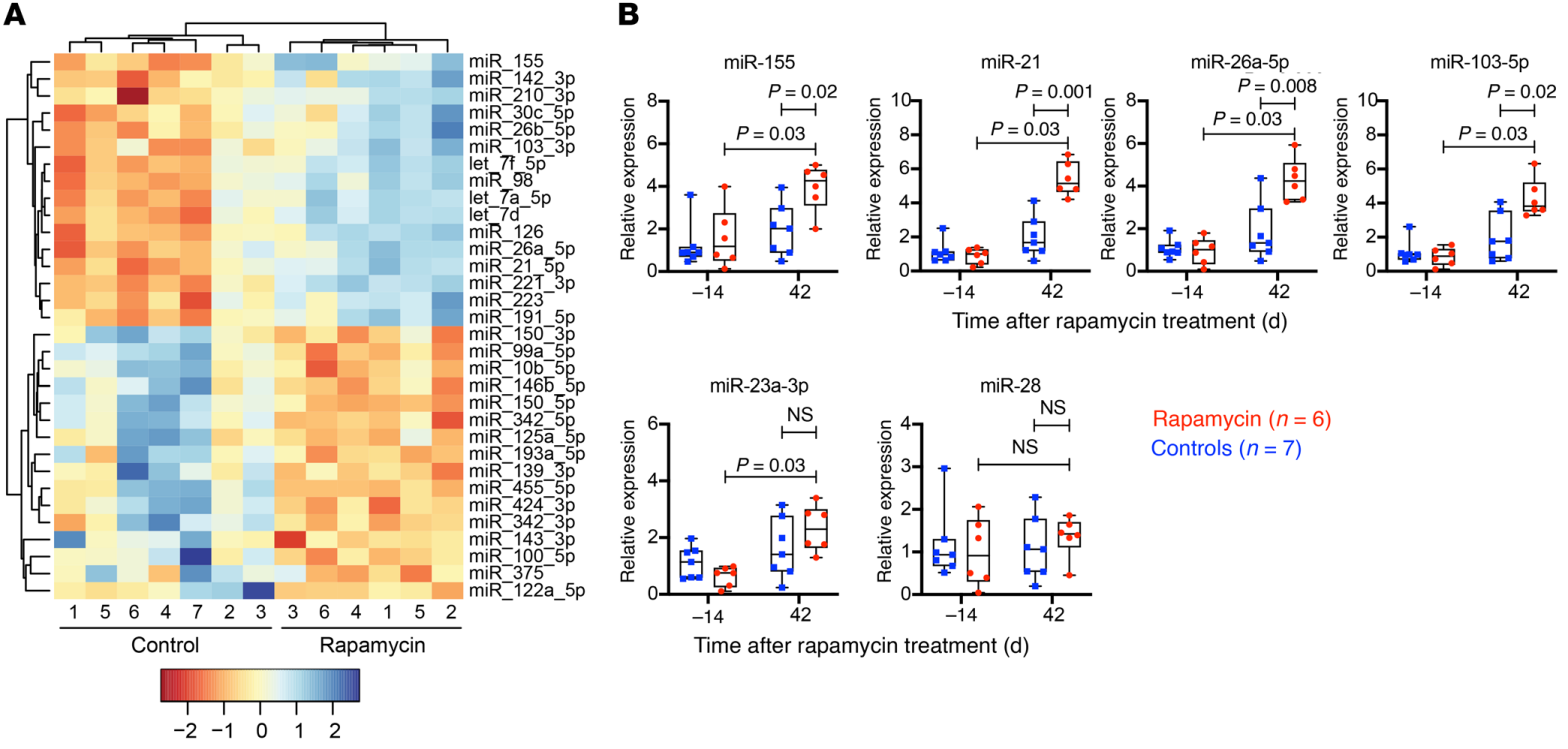
Effect of rapamycin treatment on miRNAs in plasma. (**A**) Heatmap of significant differentially expressed miRNAs in the plasma between rapamycin-treated RMs (*n* = 6) and vehicle controls (*n* = 7) after 42 days of treatment. For these analyses, *n* = 13; miRNA sequencing libraries for 1 animal in the rapamycin group failed initial quality control steps (low read depth) and were therefore not included in the final analysis. (**B**) TaqMan qRT-PCR analysis of the indicated miRNA in plasma of rapamycin-treated RMs (*n* = 7) versus vehicle controls (*n* = 7) at –14 and 42 days after treatment. The WRS test was used to determine significance (*P* values ≤ 0.05 are shown). Box plots show jittered points, a box from 1st to 3rd quartiles (IQR), and a line at the median, with whiskers extending to the farthest data point within 1.5× IQR above and below the box.

miRNAs are approximately 22-nucleotide small noncoding RNAs that posttranscriptionally regulate a variety of genes including those associated with immune cell development and cell proliferation (51). miRNAs are known to regulate mTOR signaling through direct targeting of mTOR gene expression (52, 53). Dysregulations in miRNA expression have been linked to disease progression, particularly in cancer, and as such, miRNAs represent attractive candidates as minimally invasive biomarkers for diagnostic and/or prognostic purposes (54). To further assess the systemic impact of rapamycin on mTOR-signaling regulation, we profiled miRNA expression in the plasma after 6 weeks of treatment using high-throughput sequencing. Differentially expressed miRNAs, comparing day –14 to day 42 of rapamycin treatment, were determined based on fold changes (>2-fold) and *P* values (*P* < 0.01). Significant differences in circulating miRNAs between rapamycin-treated RMs and controls were observed (Figure 2A and Supplemental Figure 3A). In particular, a number of miRNAs associated with mTOR were upregulated in response to rapamycin treatment, including miR-155, miR-21, and miR-126, which directly target core components of the mTOR-signaling pathway (53, 55–58). miR-126 suppresses expression of TSC1, a negative regulator of mTOR (57), while miR-21 targets PTEN, promoting cell growth (53, 58). Notably, miR-155 plays a key role in immune homeostasis and can promote HIV latency by suppressing the tripartite motif-containing protein 32 (TRIM32), an E3 ubiquitin ligase that activates NF-κB (59). miR-99a and miR-150, both of which cooperatively repress mTOR signaling to promote CD4^+^ Treg differentiation (60), were downregulated in response to rapamycin (Figure 2A and Supplemental Figure 3A). Other miRNAs involved with either suppressing or activating the mTOR pathway, including miR-26a, miR-122, miR-125a, miR-193a-5p, and miR-221, were also affected by rapamycin treatment (52, 53, 61).

To independently confirm differential miRNA expression, we used TaqMan-based miRNA stem-loop quantitative reverse-transcription PCR (qRT-PCR) assays to quantitate 4 miRNAs (miR-155, miR-21, miR-26a, and miR-103-5p) that were upregulated in rapamycin-treated RMs relative to controls and 2 miRNAs (miR-23a-3p and miR-28) that were unchanged between treatment groups. As expected, expression levels determined by qRT-PCR for these miRNAs recapitulated their expression patterns observed by deep sequencing (Figure 2B). Of note, miR-26a-5p and miR-155 were commonly upregulated in both treatment groups after 42 days of rapamycin or vehicle control treatment; however, their levels were significantly higher in rapamycin-treated RMs compared with controls (Figure 2B and Supplemental Figure 3, B and C). In contrast, miR-23a-3p was also upregulated by day 42 after rapamycin or vehicle control treatment, but its levels were not significantly different between treatment groups. Finally, we performed a correlative analysis to determine the relationship between miRNA expression and CD4^+^ T cell dynamics or cell-associated viral loads. Strikingly, levels of miR-155, miR-21, miR-26a, and miR-103-5p significantly correlated (*P* < 0.05) with frequencies of proliferating CD4^+^ memory T cells (TM cells) in blood (Supplemental Figure 4, A and B). Collectively, these data are consistent with previous observations showing rapamycin can substantially reprogram miRNA expression (53, 62), which has implications for cell survival, metabolism, and cell-fate decisions regarding immune responses.

### Effects of rapamycin on global gene expression.

**Figure 3:**
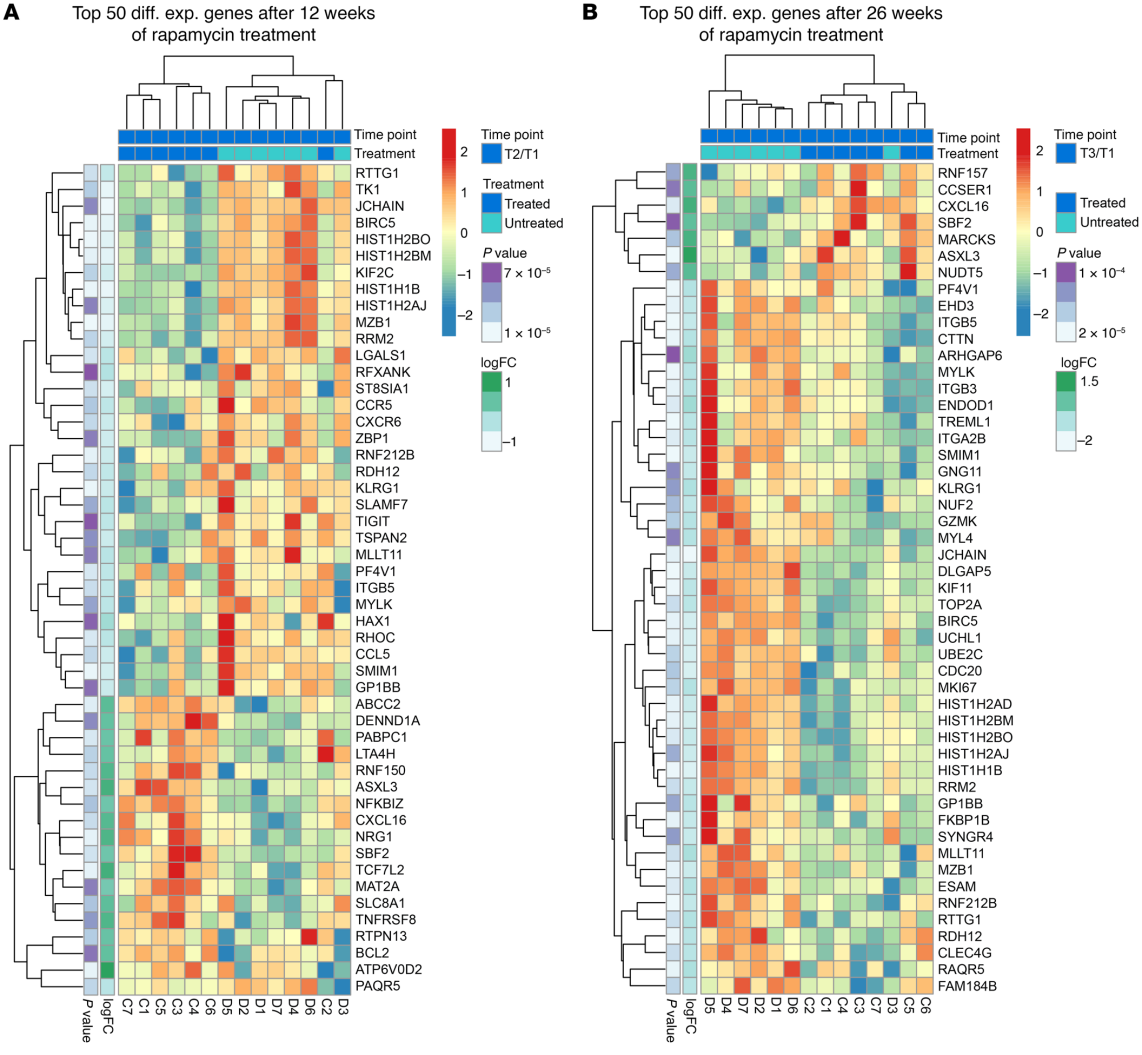
Effect of rapamycin treatment on global gene expression. (**A**) Heatmaps of the top 50 differentially expressed genes following 12 weeks of rapamycin. (**B**) Heatmaps of the top 50 differentially expressed genes following 26 weeks of rapamycin. Rapamycin-treated RMs are indicated as treated in dark blue (*n* = 7), while vehicle control RMs are indicated as untreated in light blue (*n* = 7).

To further characterize the effects of rapamycin, we performed gene-expression profiling on whole blood at 12 and 26 weeks of rapamycin treatment. We identified 881 and 358 differentially expressed genes at 12 and 26 weeks, respectively, with nominal *P* values less than or equal to 0.05 (FDR < 0.25) in rapamycin-treated RMs relative to controls. Analysis of the top 50 differentially expressed genes visualized as heatmaps in Figure 3, A and B, revealed rapamycin downregulated several histone genes (e.g., *HIST1H2BO*, *HIST1H2BM*, *HIST1H1B*, and *HIST1H2AJ*) and the baculoviral inhibitor of apoptosis repeat-containing 5 (*BIRC5*), also known as Survivin. Expression of thymidine kinase 1 (*TK1*), a cytosolic enzyme involved with cellular proliferation, was also decreased in rapamycin-treated RMs (63). Rapamycin also downregulated genes involved with cell trafficking, including the HIV and SIV entry coreceptors *CCR5* and *CXCR6* as well as *CCL5*, also known as *RANTES*. In contrast, expression of *CXCL16*, a chemokine that interacts with *CXCR6*, was increased with rapamycin treatment. A complete list of genes differentially expressed between rapamycin-treated RMs and controls is provided in Supplemental Table 2. Of note, reductions in the numbers of differentially expressed genes between weeks 12 and 26 suggest a decrease in the efficacy of mTOR inhibition with rapamycin over time.

Analysis of the top differentially enriched KEGG pathways revealed rapamycin altered numerous signaling pathways after both 12 and 26 weeks of ongoing treatment. Pathways associated with cell division (e.g., DNA replication, base excision repair, mismatch repair, and homologous recombination pathways) as well as cell growth and metabolism (e.g., citrate acid cycle, riboflavin metabolism, purine metabolism, glyoxylate and dicarboxylate metabolism, glycine, serine, and threonine metabolism) were downregulated in rapamycin-treated RMs (Supplemental Figure 5, A and B). Finally, expression of pathways involved with insulin signaling and type II diabetes mellitus were increased with rapamycin treatment, further confirming the important role of mTOR in regulating glucose metabolism and insulin sensitivity.

### Effects of rapamycin on T cell homeostasis.

**Figure 4:**
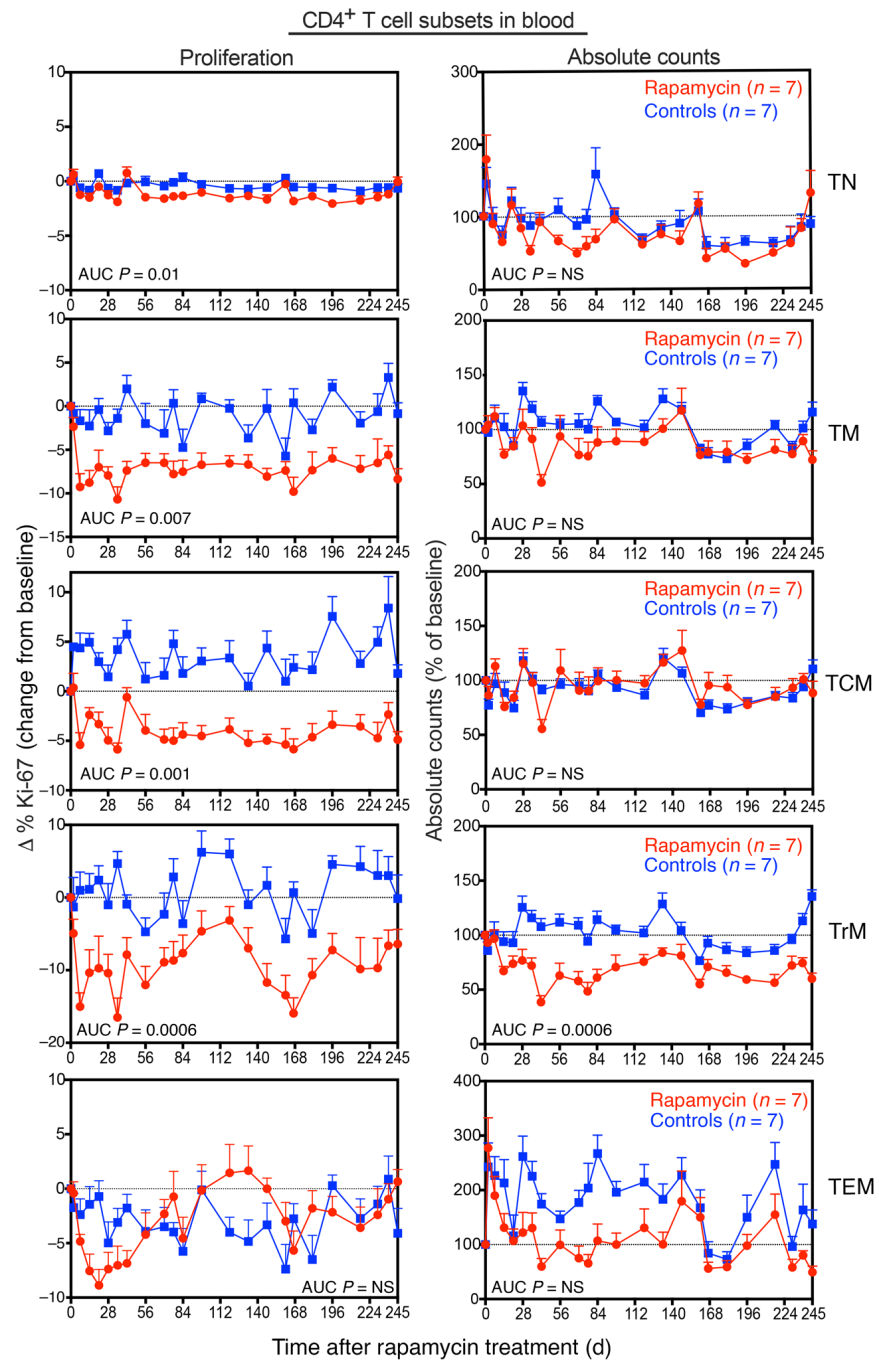
Effect of rapamycin treatment on CD4^+^ T cell subset dynamics in blood. Change in the proliferative fraction (left panels) and absolute counts (right panels) of CD4^+^ T subsets, including TN cells, TM cells, TCM cells, TrM cells, and TEM cells in blood following rapamycin (*n* = 7) versus vehicle control (*n* = 7) treatment. Results are shown as mean (+SEM) change from baseline of percentages of Ki-67 (left panels) and percentages of baseline absolute counts (right panels). The WRS test was used to determine the significance of differences in AUC between the 2 treatment groups (*P* values ≤ 0.05 are shown).

Next, we used flow cytometry to explore the effects of mTOR inhibition with rapamycin on T cell dynamics in blood and tissues. Rapamycin treatment was associated with a significant (*P* = 0.007) decline in the fraction of proliferating CD4^+^ TM cells in blood (Figure 4 and Supplemental Figure 6). In particular, the frequencies of proliferating CD4^+^ central memory cell (TCM cell) and TrM cell subsets were significantly reduced (*P* = 0.001 and *P* = 0.0006, respectively) relative to controls. In contrast, CD4^+^ effector memory T cell (TEM cell) proliferation initially declined, but gradually normalized to levels similar to those of controls by day 56 of rapamycin treatment. In LNs and BM, there were similar decreases in the fraction of proliferating CD4^+^ TM cells (*P* = 0.04), which was mostly associated with significant decreases in proliferating CD4^+^ TCM cells in both tissues (LN, *P* = 0.04; BM, *P* = 0.02; Supplemental Figure 7). Absolute CD4^+^ T cell counts also declined following rapamycin treatment, but gradually normalized to levels observed in control RMs in all subsets except CD4^+^ TrM cells, which remained markedly decreased for up to 35 weeks after rapamycin treatment (Figure 4 and Supplemental Figure 8).

**Figure 5:**
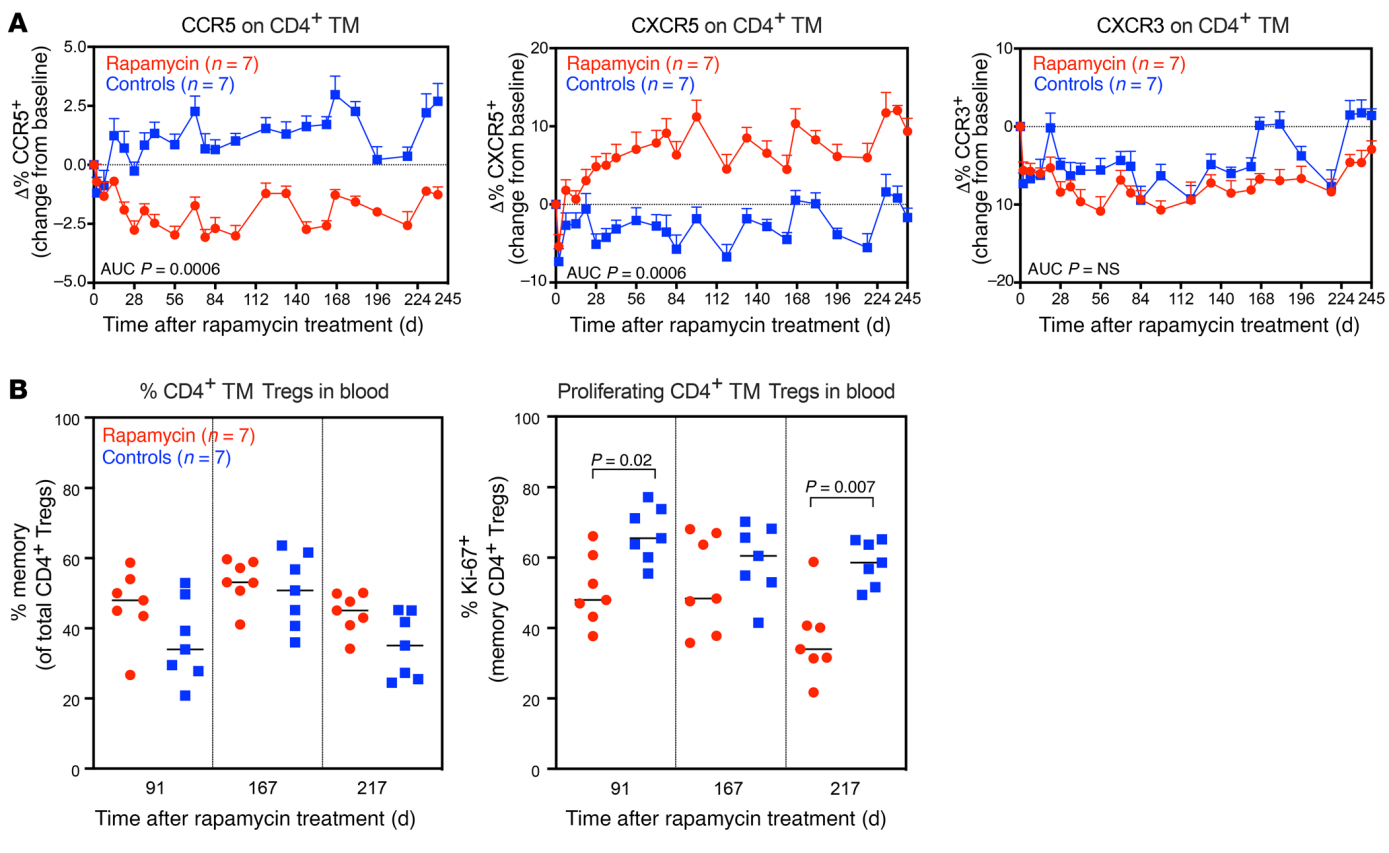
Effect of rapamycin treatment on CD4^+^ TM cell polarization. (**A**) Mean (+SEM) change from baselines of percentages of CCR5, CXCR5, and CXCR3 on CD4^+^ TM cells in blood of rapamycin-treated RMs (*n* = 7) versus vehicle controls (*n* = 7). (**B**) Comparison of percentages of CD4^+^ memory Tregs (left panel) and percentages of Ki-67^+^CD4^+^ memory Tregs (right panel) in blood of rapamycin-treated RMs (*n* = 7) versus vehicle controls (*n* = 7) during ART. Each data point represents a single determination from an individual RM. The WRS test was used to determine the significance of differences in AUC between the 2 treatment groups (*P* values ≤ 0.05 are shown).

Consistent with the gene-expression data and as previously reported (30), rapamycin reduced the frequency of CCR5^+^CD4^+^ TM cells in blood (Figure 5A and Supplemental Figure 6). While levels of CXCR3^+^CD4^+^ TM cells were similar between treatment groups, rapamycin induced a substantial increase in the frequency of CXCR5^+^CD4^+^ TM cells. We then assessed the impact of rapamycin on markers of CD4^+^ T cell activation and immune exhaustion, including CD69, CD25, HLA-DR, and PD-1. We saw no substantial difference in levels of CD69 and CD25 on CD4^+^ TM cells in blood between rapamycin-treated RMs and controls (Supplemental Figure 9). This is generally consistent with observations in PWH who received the mTOR inhibitor everolimus (35). However, there was some reduction in HLA-DR^+^ and PD-1^+^ on CD4^+^ TM cells during the first 14 weeks of rapamycin treatment, but this was not sustained long term. We also quantified the frequencies of CD4^+^ memory Tregs in blood at 91, 167, and 217 days after rapamycin treatment. This was based on previous reports suggesting rapamycin can induce Treg expansion (64–67). We found increased frequencies of CD4^+^ memory Tregs in the blood of rapamycin-treated RMs relative to controls, but this difference did not achieve statistical significance (Figure 5B). However, we did observe a marked reduction in the fraction of proliferating CD4^+^ memory Tregs at days 91 and 217 after rapamycin treatment.

**Figure 6:**
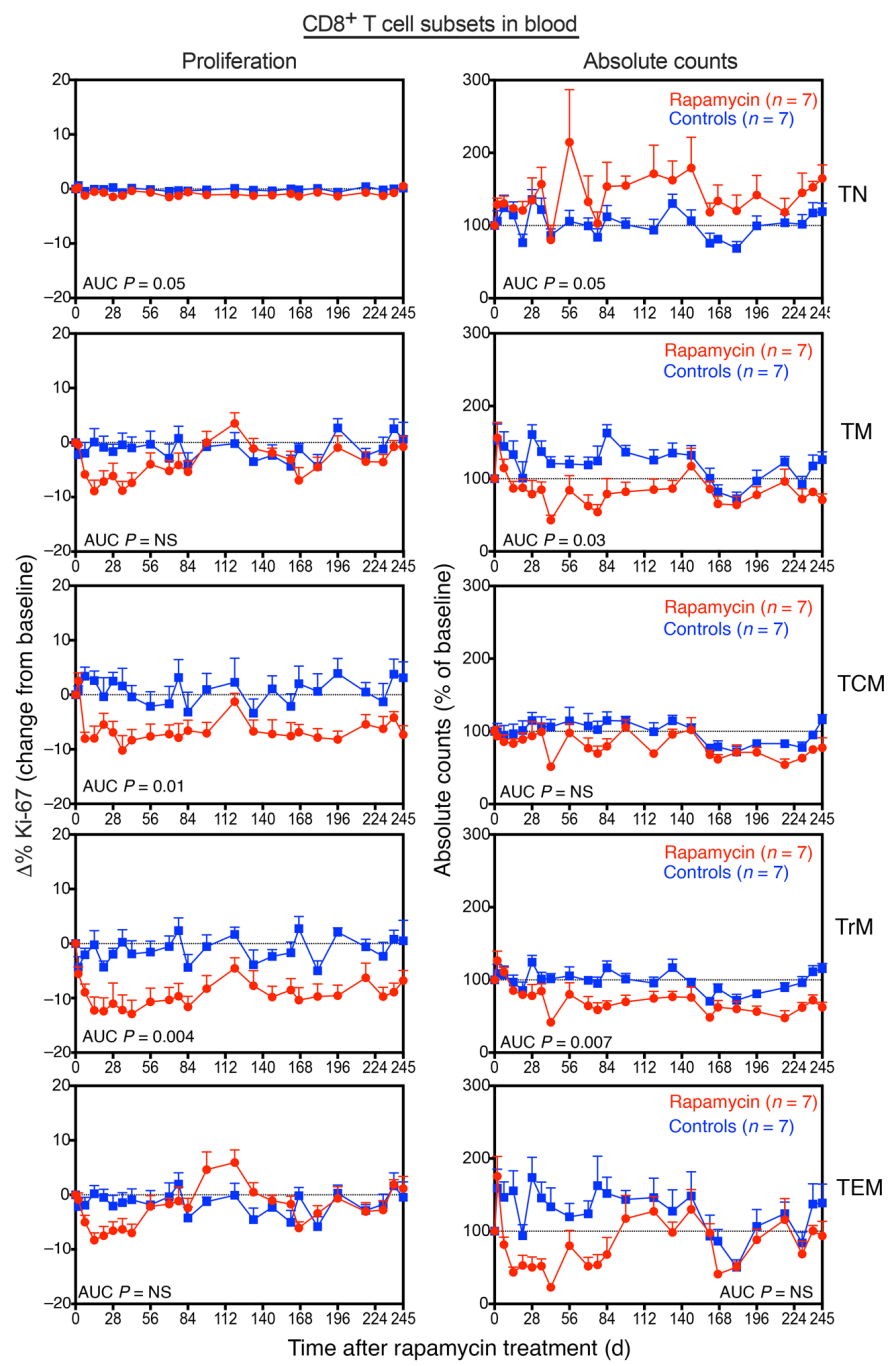
Effect of rapamycin treatment on CD8^+^ T cell subset dynamics in blood. Change in the proliferative fractions (left panels) and absolute counts (right panels) of CD8^+^ T subsets, including TN cells, TM cells, TCM cells, TrM cells, and TEM cells in blood following rapamycin (*n* = 7) versus vehicle control (*n* = 7) treatment. Results are shown as mean (+SEM) change from baseline of percentages of Ki-67 (left panels) and percentages of baseline absolute counts (right panels). The WRS test was used to determine the significance of differences in AUC between the 2 treatment groups (*P* values ≤ 0.05 are shown).

Rapamycin also affected CD8^+^ T cell homeostasis, with the fraction of proliferating CD8^+^ TCM and TrM cells in blood substantially reduced in rapamycin-treated RMs relative to controls (Figure 6). There was a reduction in CD8^+^ T cell counts in rapamycin-treated RMs (Supplemental Figure 8), in particular, CD8^+^ TrM cell counts decreased throughout the treatment period, while CD8^+^ TEM counts initially declined, but gradually normalized after 20 weeks of rapamycin treatment (Figure 6). In contrast to CD4^+^ TM cells, frequencies of CXCR3^+^ and CXCR5^+^CD8^+^ TM cells were not different between rapamycin-treated RMs and controls (Supplemental Figure 10). Rapamycin also had an effect on B cell dynamics, as absolute counts and frequencies of proliferating CD20^+^ B cells were lower in rapamycin-treated RMs relative to controls (Supplemental Figure 11A). In contrast, rapamycin had a negligible impact on NK cell dynamics, with only the CD16^–^CD56^–^ NK cell subset showing a marked decrease in counts (Supplemental Figure 11B). Collectively, these data demonstrate the profound but differential effects of rapamycin treatment on immune cell homeostasis. Interestingly, despite rapamycin’s immunomodulatory effects, we saw no evidence of increased susceptibility to AIDS-associated γ-herpesviruses. Indeed, we quantified levels of rhesus lymphocryptovirus (LCV) and rhesus rhadinovirus (RRV) in blood, both of which are oncogenic γ-herpesviruses that are simian homologues of EBV and Kaposi’s sarcoma–associated herpesvirus (KSHV), respectively, that have been associated with tumor development in the setting of impaired immunity (68–71). LCV loads remained stable through the course of rapamycin treatment, while no RRV viremia was observed in blood (Supplemental Figure 12). However, as we did not characterize virus-specific CD4^+^ and CD8^+^ T cell responses, the full immunomodulatory effects of rapamycin on T cell immunity in SIV-infected RMs on ART remain incompletely defined.

### Effects of rapamycin on SIV dynamics.

**Figure 7:**
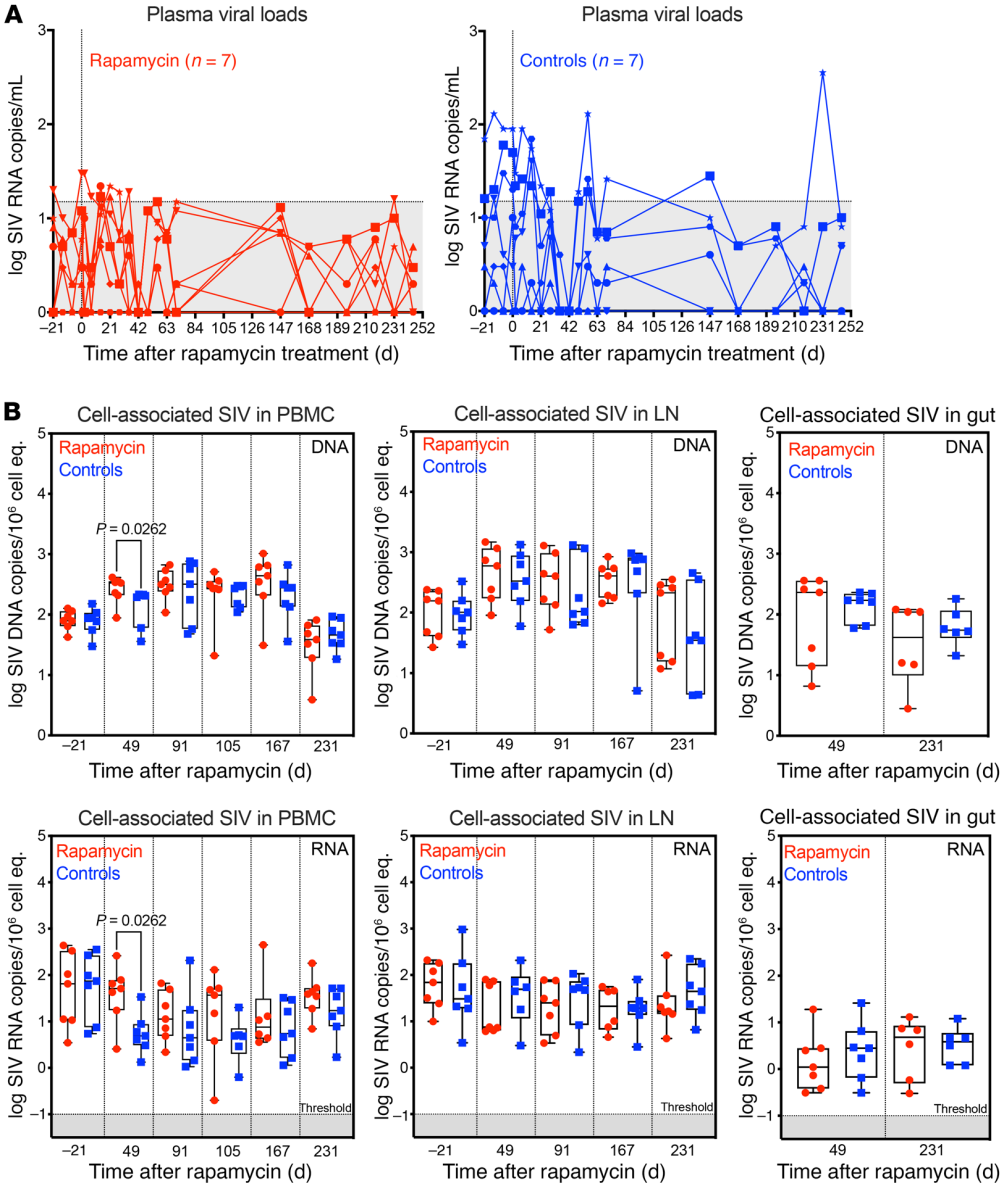
Effect of rapamycin on SIV dynamics during ART. (**A**) Individual pvl profiles monitored by a high-sensitivity assay (limit of detection [LOD] of 1 RNA copy/ml) prior to and during rapamycin (*n* = 7) or vehicle control treatment (*n* = 7), prior to ART cessation. The area in gray denotes pvl values below threshold of the standard assay (15 RNA copies/ml). (**B**) Comparison of cell-associated SIV DNA (top panels) and RNA (bottom panels) in PBMCs, LNs, and gut (copies per 10^6^ cell equivalents) after 231 days of treatment with rapamycin (*n* = 7) or vehicle control (*n* = 7). Each data point represents a single determination from an individual RM. Plots show jittered points with a box from 1st to 3rd quartiles (IQR) and a line as the median, with whiskers extending to the farthest data point within 1.5× IQR above and below the box, respectively. For **B**, the WRS test was used to determine the significance of differences between treatment groups (*P* values ≤ 0.05 are shown.

Plasma SIV RNA was monitored at least weekly 7 days prior to and during rapamycin treatment to look for any treatment-related effects on plasma viremia. Overall, there was no significant difference in the number of viral blips (i.e., measured plasma viremia above 15 SIV RNA copies/mL in ART-suppressed RMs) among rapamycin-treated RMs and controls over the first 35 weeks of observation on ART (Figure 7A). Note that 2 of 7 control RMs and 1 of 7 RMs in the rapamycin-treatment group showed 1 viral blip above the standard threshold of 15 SIV RNA copies per ml after day 147, in general indicating that mTOR inhibition with rapamycin had no consistent effect of raising levels of residual viremia during ART. Although there was a significant increase in levels of cell-associated SIV DNA and SIV RNA in PBMCs on day 49 after rapamycin, possibly due to transient lymphocyte redistribution, at all other time points, the levels of SIV RNA and SIV DNA were not statistically different between rapamycin-treated RMs and controls in PBMCs, LNs, or gut (Figure 7B). Note that when RMs are treated with ART within 1 month of SIV infection, the vast majority of cell-associated SIV DNA that persists after more than 9 months of ART contains intact genomes (72). Taken together, these data suggest that levels of persistently infected cells were largely unchanged with rapamycin treatment.

**Figure 8:**
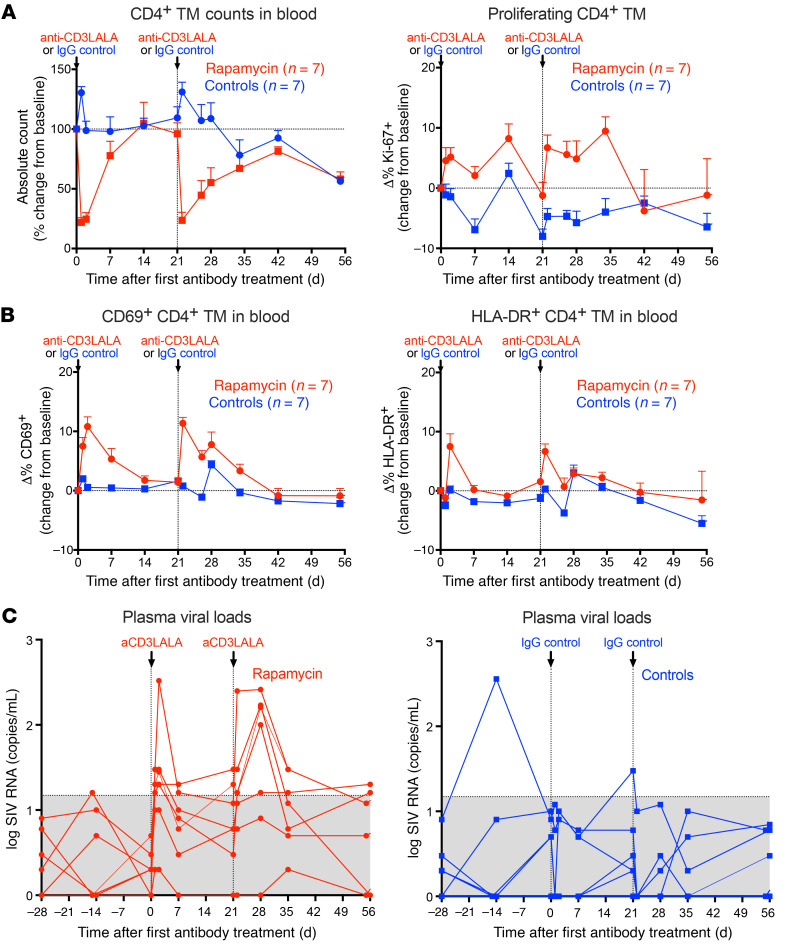
Effect of anti-CD3LALA with rapamycin on SIV dynamics during ART. (**A**) Mean (+SEM) change from baseline of percentages of absolute counts (left panel) and percentages of Ki-67 (right panel) of CD4^+^ TM cells in blood following infusion of anti-CD3LALA at 0.5 mg/kg in rapamycin-treated RMs (*n* = 7) versus 0.5 mg/kg of IgG isotype control mAbs in vehicle control–treated RMs (*n* = 7). (**B**) Mean (+SEM) change from baseline of percentages of CD69 (left panel) and percentages HLA-DR (right panel) on CD4^+^ TM cells in blood. (**C**) Individual pvl profiles monitored by a high-sensitivity assay (LOD of 1 RNA copy/ml) following anti-CD3LALA infusion in rapamycin-treated RMs (*n* = 7) or IgG isotype control mAbs in vehicle control–treated RMs (*n* = 7) during ART. The area in gray denotes pvl values below threshold of the standard assay (15 RNA copies/ml). WRS test was used to determine the significance of differences (**A** and **B**, peak increase or decrease from baseline: 2 1-sided tests; **C**, number of blips above threshold: 2-sided test) between treatment groups.

Having observed no long-term effect of rapamycin on SIV dynamics during ART, we explored whether rapamycin could be used in combination with a potent activating T cell receptor agonist to induce SIV gene expression from latently infected cells and facilitate SIV reservoir reduction. We administered 2 doses at 3-week intervals of a rhesus anti-CD3LALA mAb that incorporates the L234A and L235A mutations in the Fc receptor binding site (73), which effectively eliminates Fc-dependent in vivo target cell depletion. Anti-CD3LALA was given at 0.5 mg/kg to rapamycin-treated RMs starting 245 days following the start of rapamycin treatment. Due to concerns for potential adverse effects with administration of anti-CD3LALA in the absence of concomitant rapamycin treatment, the control group received a rhesus isotype control mAb at the same dosing schedule as anti-CD3LALA. The combination of rapamycin and anti-CD3LALA was well tolerated, with no adverse clinical events observed. This supports previous data suggesting rapamycin may dampen the levels of proinflammatory cytokines produced in response to massive T cell activation (74). Anti-CD3LALA induced a significant decrease in CD4^+^ TM cells in blood (change from baseline, *P* = 0.0005), but counts generally recovered after 2 weeks (Figure 8A). This transient decline in counts is likely driven by a redistribution of cells from blood to tissues following T cell activation in vivo. A similar effect was recently observed following infusion of an ingenol-based protein kinase C agonist in RMs (75). Following each dose of anti-CD3LALA, there was a significant increase in proliferating CD4^+^ TM cells (% Ki-67^+^ cells change from baseline, *P* = 0.006) that peaked at approximately 10% above baseline by day 14. In addition, CD69 and HLA-DR were also upregulated on CD4^+^ TM cells (change from baseline, *P* = 0.0005 and *P* = 0.01, respectively) following each infusion of anti-CD3LALA (Figure 8B). Strikingly, there was a significant increase in viral blips in plasma (*P* = 0.006) following anti-CD3LALA infusion. Four RMs showed greater than 2 log increases in SIV RNA in plasma after 1 or both doses of anti-CD3LALA, suggesting an increase in SIV gene expression (Figure 8C). Analysis of cell-associated SIV RNA and SIV DNA in PBMCs showed variable changes in levels between days 0 and 7 following each anti-CD3LALA infusion in rapamycin-treated RMs (Supplemental Figure 13).

**Figure 9:**
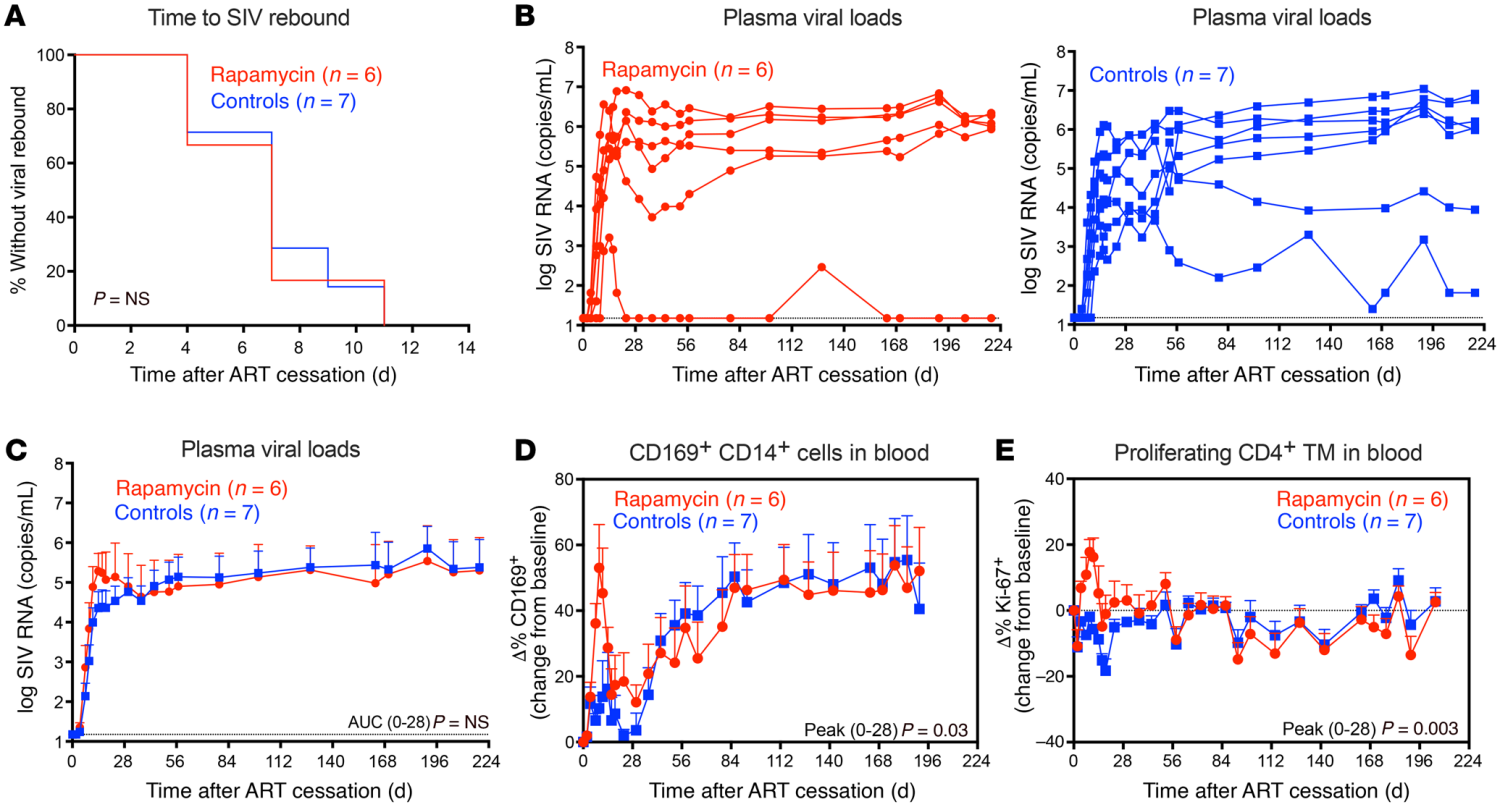
Effect of rapamycin on SIV infection dynamics after ART withdrawal. (**A**) Kaplan-Meier analysis of SIV rebound kinetics in RMs treated with rapamycin (red; *n* = 6) versus vehicle controls (blue; *n* = 7). For these analyses, *n* = 13; 1 animal in the rapamycin group was lost from study just prior to ART withdrawal and was therefore not included in the final analysis. (**B**) Individual pvl profiles of RMs in each treatment group. Left panel shows rapamycin-treated RMs (blue), while right panel shows vehicle controls (blue). (**C**) Mean (+SEM) pvl profiles of RMs stratified by treatment group (LOD; 15 RNA copies/ml). WRS test was used to determine significance of differences in the AUC of pvl. (**D**) Mean (+SEM) change from baselines of percentages of CD169 in blood of rapamycin-treated RMs (*n* = 6) versus vehicle controls (*n* = 7) following ART withdrawal. (**E**) Mean (+SEM) change from baselines of percentages of Ki-67 in blood of rapamycin-treated RMs (*n* = 6) versus vehicle controls (*n* = 7) following ART withdrawal. The WRS test was used to determine the significance of differences between treatment groups (days 0–28 AUC or peak; *P* values ≤ 0.05 are shown).

After a 6-week washout period following the last dose of anti-CD3LALA, both ART and rapamycin were discontinued to assess the combined effects of mTOR inhibition and T cell activation on SIV rebound dynamics. Just prior to ART release, 1 RM in the rapamycin treatment group was lost from the study due to unrelated health complications. Of the remaining RMs, all manifested viral rebound within 12 days of ART release with no significant differences in the time to measurable rebound viremia between rapamycin-treated RMs and controls (Figure 9A). ART withdrawal was associated with a rapid increase in pvl, which peaked at a mean of 5.3 logs in rapamycin-treated RMs in comparison with 4.4 logs in controls by day 14 after ART (Figure 9, B and C). However, both peak pvl and early viral burden (as measured by the AUC of pvl 0–28 days after ART) were similar between treatment groups. SIV replication in rapamycin-treated RMs subsequently normalized to levels observed in control RMs by 6 weeks after ART, with both treatment groups establishing similar pvl set points. This suggests that rapamycin treatment with T cell activation had no long-term effect on SIV-specific immunity. Indeed, antigen-driven expansion of SIV-specific CD8^+^ T cells in response to rebound viremia was similar between rapamycin-treated RMs and controls (Supplemental Figure 14). Of note, ART and rapamycin withdrawal was also associated with a rapid but transient increase in monocyte activation (Figure 9D), as measured by CD169 on CD14^+^ monocytes (76, 77), and a concomitant increase in CD4^+^ TM cell proliferation immediately following treatment interruption (Figure 9E). Similar increases in proliferating CD8^+^ TM cells, B cells, and NK cells were also observed in rapamycin-treated RMs (Supplemental Figure 15, A–C). This increase in immune cell activation and proliferation could account for the early increase in peak rebound pvl observed in rapamycin-treated RMs. However, lymphocyte subset dynamics in rapamycin-treated RMs normalized to that of control RMs by 6 weeks after ART, suggesting the effect of immediate rapamycin and ART withdrawal was transient and did not affect post-ART pvl set points.

## Discussion

The pool of latently infected CD4^+^ T cells with intact HIV proviruses that persist despite ART is a major barrier to more definitive treatment of HIV infection that might be capable of resulting in either a functional or eradicative “cure.” This pool of latently infected cells is maintained by mechanisms that allow them to proliferate and expand in response to regulatory cytokines and/or persistent antigen exposure. However, proliferative expansion of latently infected cells can occur without the induction of HIV gene expression, preventing immune recognition and clearance by antiviral effector mechanisms. This raises an important question as to whether therapeutically limiting cellular proliferation could be an effective strategy for restricting the expansion of latently infected CD4^+^ T cells and facilitating the decay of the HIV reservoir (4, 17).

We used a nonhuman primate (NHP) model of ART-suppressed SIV infection to evaluate the effects of the antiproliferative agent rapamycin on viral dynamics. Rapamycin was administered to SIV-infected RMs on ART for up to 44 weeks. Chronic mTOR inhibition with rapamycin was generally well tolerated in RMs at the target dose achieved, was stably maintained, and recapitulated effects previously reported in mice and humans, including disruptions in lipid metabolism and alterations in the metabolic hormones commonly regulated by mTOR signaling. We also observed reduced expression of Glut1, which is upregulated on T cells and monocytes following HIV infection and is not completely normalized by ART (78–80). Gene-expression profiling showed rapamycin downregulated genes associated with cell division, growth, and metabolism and induced significant changes in circulating miRNAs, highlighting the multifactorial role of mTOR signaling in a host of biological processes. Interestingly, rapamycin has been shown to dramatically increase life span in a wide variety of rodent model systems (81). However, the utility of rapamycin to extend life span in other preclinical animal models has yet to be fully explored. Here, we provide for the first time, to our knowledge, a comprehensive assessment of the impact of chronic rapamycin administration on metabolism, gene expression, and immune cell homeostasis in a clinically relevant NHP model of ART-suppressed SIV infection.

In RMs, the antiproliferative properties of rapamycin were demonstrated by the substantial reductions in proliferating T cell subsets observed in blood and tissues of rapamycin-treated RMs relative to controls. Reductions in CD4^+^ and CD8^+^ T cell counts were also observed, but this was not sustained long term. While the exact reasons for this are unclear, the induction of compensatory signaling pathways in response to prolonged mTOR inhibition may have played a role (82–84). We also observed modest effects on activating and inhibitory receptors on CD4^+^ TM cells in blood despite marked decreases in proliferation. This would suggest that proliferation inhibition with rapamycin may be intrinsic and not dependent on activating or inhibitory receptors. Rapamycin also increased frequencies of CD4^+^ TM cells in blood that expressed CXCR5, the chemokine receptor associated with homing to B cell follicles, although we did not observe substantial differences in frequencies of CD4^+^ follicular helper T cells (TFH cells) in LNs of rapamycin-treated RMs relative to controls (data not shown). However, the rapamycin-induced increase in circulating TFH cells might provide clues as to why mTOR inhibition has been shown to improve vaccine outcomes in older adults (85, 86). Indeed, mTOR inhibition may facilitate TFH cell differentiation and trafficking following antigen exposure. Rapamycin also decreased expression of the HIV/SIV entry coreceptor CCR5 on CD4^+^ TM cells, potentially increasing their resistance to infection. In particular, we observed a prolonged reduction in CCR5^hi^CD4^+^ TrM cells in rapamycin-treated RMs, likely due to rapamycin downregulation of CCR5 expression. Despite these profound changes in CD4^+^ T cell dynamics, analysis of various virologic parameters, including SIV RNA in plasma and cell-associated viral loads in PBMCs and tissues, indicated no substantial impact of rapamycin on SIV persistence in RMs on ART. In addition, T cell activation with 2 doses of anti-CD3LALA did not affect SIV DNA levels in RMs on rapamycin or time to SIV rebound following ART cessation.

Possible explanations as to why rapamycin had no significant impact on SIV dynamics in our study include the following: (a) although rapamycin was able to significantly reduce the proliferative rates of CD4^+^ TM cells, this reduction was not of sufficient magnitude to affect SIV reservoir stability. Mathematical modeling suggested that even a 2.2-fold reduction in CD4^+^ T cell proliferation for more than 17 weeks may induce a 10-fold reduction in the HIV reservoir (17). However, the optimal threshold by which proliferation must be reduced in order to accelerate the decay of persistently infected cells is still unclear. (b) While rapamycin had a substantial effect on CD4^+^ TCM and TrM cell proliferation, populations that typically maintain higher rates of proliferation, it had less of an impact on CD4^+^ naive (TN) and TEM cells, which tend to be more quiescent and proliferate at lower rates under steady-state conditions. However, these subsets can also contain intact HIV proviruses that can contribute to HIV persistence (87, 88). Indeed, this highlights a major challenge with targeting proliferation as a curative strategy for HIV cure, as antiproliferative agents such as rapamycin may be less effective against resting/quiescent cell subsets harboring intact HIV proviruses. (c) The duration of rapamycin treatment (i.e., 44 weeks) may not have been sufficient to significantly affect SIV dynamics during ART. In PWH, the reservoir decays slowly, with the half-life of infected cells estimated to be approximately 44 months (89). This slow rate of decay suggests antiproliferative therapies may need to be applied for extended periods of time in order to have a measurable impact on the reservoir. (d) The role of homeostatic proliferation in maintaining latently infected CD4^+^ T cells in different tissue compartments is still unclear. For example, a high proportion of latently infected cells during ART reside in gut-associated lymphoid tissues (90, 91). These cells can often be terminally differentiated with limited proliferative capacity and may be less susceptible to the factors that drive cytokine and/or antigen-driven clonal expansion of latently infected cells in peripheral sites (92).

Our study did result in 2 interesting observations. First, the use of a potent T cell receptor agonist in the presence of rapamycin had no toxic side effects in SIV-infected RMs on ART, despite clear evidence of induction of T cell proliferation. Even at a low dose of 0.5 mg/kg, 4 of 7 RMs had a modest increase in plasma viremia following anti-CD3LALA infusion that was consistent with increased SIV production. Previous attempts to use a potent T cell agonist to induce HIV production in vivo involved the use of a murine anti-CD3 mAb, OKT3, given in combination with recombinant IL-2 (93, 94). This therapy induced T cell activation and proliferation in addition to some measurable increases in HIV RNA in both plasma and LNs of PWH. However, this therapy had toxic side effects and subsequently diminished enthusiasm for the application of T cell agonists as LRAs. Our results suggest that rapamycin, or other mTOR inhibitors, used in combination with potent T cell receptor agonists may be an effective strategy for limiting CD4^+^ T cell proliferation, reducing production of proinflammatory cytokines while safely inducing HIV gene expression during ART (74). While we show a low dose of anti-CD3LALA was safe, further studies are required to assess whether mTOR inhibition can still limit toxicity when anti-CD3LALA is used repeatedly or at higher doses. It should also be noted that the introduction of the LALA mutations on the anti-CD3 antibody prevents the crosslinking of Fc receptors on T cells and Fc-dependent target cell depletion, which may have also reduced toxicity following T cell activation (95, 96).

The other intriguing observation was the rapid increase in early peak rebound pvl, likely driven by the transient increase in T cell proliferation immediately following ART and rapamycin treatment cessation. This occurred at a time when rebounding viremia would be inducing antigen-driven expansion of SIV-specific T cells (40). The increased rate of T cell proliferation may have been associated with changes in cellular metabolism, as metabolic reprograming by rapamycin has been shown to enhance the generation of antigen-specific CD8^+^ T cells (97, 98). However, similar increases in proliferating B cells and NK cells suggest that this phenomenon may not be restricted to antigen-specific T cells. An alternative explanation is that the proliferation was driven by a change in the metabolic fitness of cells as they were released from impaired nutrient sensing due to mTOR inhibition (99). In general, the increased proliferative response was transient and did not alter the trajectory of SIV replication long term, as pvl set points and post-ART pvl AUC were similar between treatment groups. Intriguingly, post-ART viremia was similar despite reductions in CCR5 expression on CD4^+^ T cells in rapamycin-treated RMs. However, this effect was rather modest (2%–3% reduction), and as we did not see a similar reduction in frequencies of CCR5^+^CD4^+^ TM cells in LNs, the observed differences might reflect changes in recirculation patterns rather than differences in total body number of SIV targets (which reside in tissues, particularly in mucosal sites). Also, rapamycin and ART were stopped concomitantly, so CCR5 expression patterns would be expected to normalize following ART cessation.

In conclusion, while proliferation of latently infected CD4^+^ T cells plays an important role in driving HIV persistence during therapy, our study highlights some major challenges with targeting this regulatory mechanism as a strategy for reducing viral reservoirs that persist during ART. Questions regarding how long antiproliferative therapies should be applied and their effectiveness on different cell subsets (i.e., naive versus memory) or in different sites (tissue resident versus circulating) would need to be addressed to fully determine whether this approach can be used to enhance the decay of the HIV reservoir in PWH.

## Methods

Detailed materials and methods can be found in Supplemental Methods.

### Statistics.

Statistical analyses were performed in R 3.6.0 with the package survival, version 3.2-11. We used Spearman’s rank transformation when evaluating correlations. We used Wilcoxon’s rank-sum (WRS) tests for all analyses comparing values across treatment groups. Point values were transformed to the log_10_ scale where indicated. For analyses involving multiple time points, we calculated the AUC, peak value, or number of “blips” (observations above a predefined threshold) for each RM and analyzed the resulting values in a fashion similar to that using single–time point data. Time-to-event data were described with Kaplan-Meier estimates and compared between groups using the Kruskal-Wallis test. All tests were conducted with 2-sided null hypotheses at a significance level of *P* ≤ 0.05, except for experiments on the effect of anti-CD3LALA (Figure 8, A and B), for which we used two separate 1-sided tests with a significance level of *P* ≤ 0.025. While we present primarily unadjusted *P* values in accordance with our prespecified plan, and consistent with our usual practice, we value and encourage consideration of the impact of multiple testing. For post hoc multiplicity adjustment over any set of tests, we provide all unadjusted *P* values in Supplemental Table 3 and Supplemental Data and Code.

### Study approval.

All animal procedures were approved by the Oregon Health & Science University, Oregon National Primate Research Center’s IACUC, under the standards of the US NIH *Guide for the Care and Use of Laboratory Animals* (National Academies Press, 2011).

## Author contributions

AAO, CMAC, and TJH obtained funding and conceived and planned the study. AAO supervised RM experiments and wrote the paper with assistance from BDVM, PTE, RLS, JDL, and LJP. WB and BDVM analyzed immunological and virological data, assisted by AM, DDD, and YF. CMAC provided RNA-Seq assays and data, while BR performed bioinformatic analysis. RLS provided miRNA and LCV quantification, assisted by DNF. SWW provided RRV quantification. JDL provided SIV quantification. JDE provided in situ Glut1 analysis, assisted by KBS. WF and KE produced and provided anti-CD3LALA. JS and MKA managed the animal protocols. PTE and DS conducted statistical analyses.

## Supplementary Material

Supplemental data

Supplemental data set 1

Supplemental table 2

Supplemental table 3

## Version 1 03/22/2022

In-Press Preview

## Version 2 05/16/2022

Electronic publication

## References

[1] Finzi D (1999). Latent infection of CD4+ T cells provides a mechanism for lifelong persistence of HIV-1, even in patients on effective combination therapy. Nat Med.

[2] Chun TW (1997). Presence of an inducible HIV-1 latent reservoir during highly active antiretroviral therapy. Proc Natl Acad Sci U S A.

[3] Siliciano JD (2003). Long-term follow-up studies confirm the stability of the latent reservoir for HIV-1 in resting CD4+ T cells. Nat Med.

[4] Reeves DB (2018). A majority of HIV persistence during antiretroviral therapy is due to infected cell proliferation. Nat Commun.

[5] Virgilio MC, Collins KL (2020). The impact of cellular proliferation on the HIV-1 reservoir. Viruses.

[6] Hosmane NN (2017). Proliferation of latently infected CD4^+^ T cells carrying replication-competent HIV-1: potential role in latent reservoir dynamics. J Exp Med.

[7] Chomont N (2009). HIV reservoir size and persistence are driven by T cell survival and homeostatic proliferation. Nat Med.

[8] Wagner TA (2014). HIV latency. Proliferation of cells with HIV integrated into cancer genes contributes to persistent infection. Science.

[9] Cohn LB (2015). HIV-1 integration landscape during latent and active infection. Cell.

[10] Maldarelli F (2014). HIV latency. Specific HIV integration sites are linked to clonal expansion and persistence of infected cells. Science.

[11] Yoon JK (2020). HIV proviral DNA integration can drive T cell growth ex vivo. Proc Natl Acad Sci U S A.

[12] Coffin JM (2021). Integration in oncogenes plays only a minor role in determining the in vivo distribution of HIV integration sites before or during suppressive antiretroviral therapy. PLoS Pathog.

[13] Simonetti FR (2021). Antigen-driven clonal selection shapes the persistence of HIV-1-infected CD4+ T cells in vivo. J Clin Invest.

[14] Mendoza P (2020). Antigen-responsive CD4+ T cell clones contribute to the HIV-1 latent reservoir. J Exp Med.

[15] Vandergeeten C (2013). Interleukin-7 promotes HIV persistence during antiretroviral therapy. Blood.

[16] Musick A (2019). HIV infected T cells can proliferate in vivo without inducing expression of the integrated provirus. Front Microbiol.

[17] Reeves DB (2017). Anti-proliferative therapy for HIV cure: a compound interest approach. Sci Rep.

[18] Ke R (2018). Determinants of the efficacy of HIV latency-reversing agents and implications for drug and treatment design. JCI Insight.

[19] Saxton RA, Sabatini DM (2017). mTOR signaling in growth, metabolism, and disease. Cell.

[20] Sarbassov DD (2006). Prolonged rapamycin treatment inhibits mTORC2 assembly and Akt/PKB. Mol Cell.

[21] Mukhopadhyay S (2016). The enigma of rapamycin dosage. Mol Cancer Ther.

[22] Armand P (2008). Improved survival in lymphoma patients receiving sirolimus for graft-versus-host disease prophylaxis after allogeneic hematopoietic stem-cell transplantation with reduced-intensity conditioning. J Clin Oncol.

[23] Dufour M (2011). Targeting the mammalian target of rapamycin (mTOR) in cancer therapy: lessons from past and future perspectives. Cancers (Basel).

[24] Liu Y (2015). mTOR signaling in T cell immunity and autoimmunity. Int Rev Immunol.

[25] Cutler C, Antin JH (2010). Sirolimus immunosuppression for graft-versus-host disease prophylaxis and therapy: an update. Curr Opin Hematol.

[26] Taylor HE (2020). mTOR overcomes multiple metabolic restrictions to enable HIV-1 reverse transcription and intracellular transport. Cell Rep.

[27] Cinti A (2017). HIV-1 enhances mTORC1 activity and repositions lysosomes to the periphery by co-opting Rag GTPases. Sci Rep.

[28] Kumar B (2017). Hyperactivation of mammalian target of rapamycin complex 1 by HIV-1 is necessary for virion production and latent viral reactivation. FASEB J.

[29] Heredia A (2003). Rapamycin causes down-regulation of CCR5 and accumulation of anti-HIV beta-chemokines: an approach to suppress R5 strains of HIV-1. Proc Natl Acad Sci U S A.

[30] Heredia A (2015). Targeting of mTOR catalytic site inhibits multiple steps of the HIV-1 lifecycle and suppresses HIV-1 viremia in humanized mice. Proc Natl Acad Sci U S A.

[31] Besnard E (2016). The mTOR complex controls HIV latency. Cell Host Microbe.

[32] Jin S (2018). TSC1 and DEPDC5 regulate HIV-1 latency through the mTOR signaling pathway. Emerg Microbes Infect.

[33] Akbay B (2020). Modulation of mTORC1 signaling pathway by HIV-1. Cells.

[34] Moranguinho I, Valente ST (2020). Block-and-lock: new horizons for a cure for HIV-1. Viruses.

[35] Henrich TJ (2021). Everolimus, an mTORC1/2 inhibitor, in ART-suppressed individuals who received solid organ transplantation: A prospective study. Am J Transplant.

[36] Di Benedetto F (2010). First report on a series of HIV patients undergoing rapamycin monotherapy after liver transplantation. Transplantation.

[37] Di Benedetto F (2011). University of Modena experience in HIV-positive patients undergoing liver transplantation. Transplant Proc.

[38] Stock PG (2014). Reduction of HIV persistence following transplantation in HIV-infected kidney transplant recipients. Am J Transplant.

[39] Okoye AA (2018). Early antiretroviral therapy limits SIV reservoir establishment to delay or prevent post-treatment viral rebound. Nat Med.

[40] Okoye AA (2021). CD8+ T cells fail to limit SIV reactivation following ART withdrawal until after viral amplification. J Clin Invest.

[41] Morrisett JD (2002). Effects of sirolimus on plasma lipids, lipoprotein levels, and fatty acid metabolism in renal transplant patients. J Lipid Res.

[42] Morrisett JD (2003). Sirolimus changes lipid concentrations and lipoprotein metabolism in kidney transplant recipients. Transplant Proc.

[43] Johnson ED (2019). The D-dimer assay. Am J Hematol.

[44] Brenchley JM (2006). Microbial translocation is a cause of systemic immune activation in chronic HIV infection. Nat Med.

[45] Babinska A (1998). Enhancement of human platelet aggregation and secretion induced by rapamycin. Nephrol Dial Transplant.

[46] Wang L (2021). mTOR regulates GPVI-mediated platelet activation. J Transl Med.

[47] Xu G (2009). Gastric mammalian target of rapamycin signaling regulates ghrelin production and food intake. Endocrinology.

[48] Singh M (2016). Effect of low-dose rapamycin on senescence markers and physical functioning in older adults with coronary artery disease: results of a pilot study. J Frailty Aging.

[49] Paschoal VA (2017). mTORC1 inhibition with rapamycin exacerbates adipose tissue inflammation in obese mice and dissociates macrophage phenotype from function. Immunobiology.

[50] Liemburg-Apers DC (2016). Acute stimulation of glucose influx upon mitoenergetic dysfunction requires LKB1, AMPK, Sirt2 and mTOR-RAPTOR. J Cell Sci.

[51] Mehta A, Baltimore D (2016). MicroRNAs as regulatory elements in immune system logic. Nat Rev Immunol.

[52] Zhang Y (2017). Emerging role of MicroRNAs in mTOR signaling. Cell Mol Life Sci.

[53] Nazari N (2021). The emerging role of microRNA in regulating the mTOR signaling pathway in immune and inflammatory responses. Immunol Cell Biol.

[54] Backes C (2016). Specific miRNA disease biomarkers in blood, serum and plasma: challenges and prospects. Mol Diagn Ther.

[55] Wang J (2013). MicroRNA-155 promotes autophagy to eliminate intracellular mycobacteria by targeting Rheb. PLoS Pathog.

[56] Martin EC (2014). microRNA regulation of mammalian target of rapamycin expression and activity controls estrogen receptor function and RAD001 sensitivity. Mol Cancer.

[57] Agudo J (2014). The miR-126-VEGFR2 axis controls the innate response to pathogen-associated nucleic acids. Nat Immunol.

[58] Meng F (2006). Involvement of human micro-RNA in growth and response to chemotherapy in human cholangiocarcinoma cell lines. Gastroenterology.

[59] Ruelas DS (2015). MicroRNA-155 reinforces HIV latency. J Biol Chem.

[60] Warth SC (2015). Induced miR-99a expression represses Mtor cooperatively with miR-150 to promote regulatory T-cell differentiation. EMBO J.

[61] Wang Z (2021). Rapamycin inhibits glioma cells growth and promotes autophagy by miR-26a-5p/DAPK1 axis. Cancer Manag Res.

[62] Totary-Jain H (2013). Reprogramming of the microRNA transcriptome mediates resistance to rapamycin. J Biol Chem.

[63] Bitter EE (2020). Thymidine kinase 1 through the ages: a comprehensive review. Cell Biosci.

[64] Battaglia M (2005). Rapamycin selectively expands CD4+CD25+FoxP3+ regulatory T cells. Blood.

[65] Valmori D (2006). Rapamycin-mediated enrichment of T cells with regulatory activity in stimulated CD4+ T cell cultures is not due to the selective expansion of naturally occurring regulatory T cells but to the induction of regulatory functions in conventional CD4+ T cells. J Immunol.

[66] Stallone G (2016). mTOR inhibitors effects on regulatory T cells and on dendritic cells. J Transl Med.

[67] Hendrikx TK (2009). Monotherapy rapamycin allows an increase of CD4 CD25 FoxP3 T cells in renal recipients. Transpl Int.

[68] Orzechowska BU (2008). Rhesus macaque rhadinovirus-associated non-Hodgkin lymphoma: animal model for KSHV-associated malignancies. Blood.

[69] Rangan SR (1986). Epstein-Barr virus-related herpesvirus from a rhesus monkey (Macaca mulatta) with malignant lymphoma. Int J Cancer.

[70] Searles RP (1999). Sequence and genomic analysis of a Rhesus macaque rhadinovirus with similarity to Kaposi’s sarcoma-associated herpesvirus/human herpesvirus 8. J Virol.

[71] Wang F (2001). A new animal model for Epstein-Barr virus pathogenesis. Curr Top Microbiol Immunol.

[72] Long S (2019). Evaluating the intactness of persistent viral genomes in simian immunodeficiency virus-infected rhesus macaques after initiating antiretroviral therapy within one year of infection. J Virol.

[73] Wines BD (2000). The IgG Fc contains distinct Fc receptor (FcR) binding sites: the leukocyte receptors Fc gamma RI and Fc gamma RIIa bind to a region in the Fc distinct from that recognized by neonatal FcR and protein A. J Immunol.

[74] Martin AR (2017). Rapamycin-mediated mTOR inhibition uncouples HIV-1 latency reversal from cytokine-associated toxicity. J Clin Invest.

[75] Okoye AA (2022). The ingenol-based protein kinase C agonist GSK445A is a potent inducer of HIV and SIV RNA transcription. PLoS Pathog.

[76] Kim WK (2015). Increased expression of CD169 on blood monocytes and its regulation by virus and CD8 T cells in macaque models of HIV infection and AIDS. AIDS Res Hum Retroviruses.

[77] van der Kuyl AC (2007). Sialoadhesin (CD169) expression in CD14+ cells is upregulated early after HIV-1 infection and increases during disease progression. PLoS One.

[78] Palmer CS (2014). Increased glucose metabolic activity is associated with CD4+ T-cell activation and depletion during chronic HIV infection. AIDS.

[79] Butterfield TR (2017). Increased glucose transporter-1 expression on intermediate monocytes from HIV-infected women with subclinical cardiovascular disease. AIDS.

[80] Palmer CS (2014). Glucose transporter 1-expressing proinflammatory monocytes are elevated in combination antiretroviral therapy-treated and untreated HIV+ subjects. J Immunol.

[81] Selvarani R (2021). Effect of rapamycin on aging and age-related diseases-past and future. Geroscience.

[82] Bailey ST (2014). mTOR inhibition induces compensatory, therapeutically targetable MEK activation in renal cell carcinoma. PLoS One.

[83] O’Reilly KE (2006). mTOR inhibition induces upstream receptor tyrosine kinase signaling and activates Akt. Cancer Res.

[84] Soares HP (2013). Different patterns of Akt and ERK feedback activation in response to rapamycin, active-site mTOR inhibitors and metformin in pancreatic cancer cells. PLoS One.

[85] Mannick JB (2014). mTOR inhibition improves immune function in the elderly. Sci Transl Med.

[86] Mannick JB (2018). TORC1 inhibition enhances immune function and reduces infections in the elderly. Sci Transl Med.

[87] Hiener B (2017). Identification of genetically intact HIV-1 proviruses in specific CD4^+^ T cells from effectively treated participants. Cell Rep.

[88] Venanzi Rullo E (2019). Genetic evidence that naive T cells can contribute significantly to the human immunodeficiency virus intact reservoir: time to re-evaluate their role. Clin Infect Dis.

[89] Crooks AM (2015). Precise quantitation of the latent HIV-1 reservoir: implications for eradication strategies. J Infect Dis.

[90] Estes JD (2017). Defining total-body AIDS-virus burden with implications for curative strategies. Nat Med.

[91] Chun TW (2008). Persistence of HIV in gut-associated lymphoid tissue despite long-term antiretroviral therapy. J Infect Dis.

[92] Telwatte S (2018). Gut and blood differ in constitutive blocks to HIV transcription, suggesting tissue-specific differences in the mechanisms that govern HIV latency. PLoS Pathog.

[93] van Praag RM (2001). OKT3 and IL-2 treatment for purging of the latent HIV-1 reservoir in vivo results in selective long-lasting CD4+ T cell depletion. J Clin Immunol.

[94] Prins JM (1999). Immuno-activation with anti-CD3 and recombinant human IL-2 in HIV-1-infected patients on potent antiretroviral therapy. AIDS.

[95] Alegre ML (1992). Effect of a single amino acid mutation on the activating and immunosuppressive properties of a “humanized” OKT3 monoclonal antibody. J Immunol.

[96] Saunders KO (2019). Conceptual approaches to modulating antibody effector functions and circulation half-life. Front Immunol.

[97] Pearce EL (2009). Enhancing CD8 T-cell memory by modulating fatty acid metabolism. Nature.

[98] Araki K (2009). mTOR regulates memory CD8 T-cell differentiation. Nature.

[99] Kim J, Guan KL (2019). mTOR as a central hub of nutrient signalling and cell growth. Nat Cell Biol.

