# Supplementary material for: Rapamycin limits CD4+ T cell proliferation in simian immunodeficiency virus–infected rhesus macaques on antiretroviral therapy
Source: J Clin Invest. 2022 May 16;132(10):e156063. doi: 10.1172/JCI156063 (PMC9106346; doi:10.1172/JCI156063)
Supplement: Supplemental data [file jci-132-156063-s076.pdf]

Supplemental Materials

Rapamycin limits CD4$^+$ T cell proliferation in simian immunodeficiency virus-infected rhesus macaques on antiretroviral therapy

Benjamin Varco-Merth, William Brantley, Alejandra Marenco, Derick D. Duell, Devin N. Fachko, Brian Richardson, Kathleen Busman-Sahay, Danica Shao, Walter Flores, Kathleen Engelman, Yoshinori Fukazawa, Scott W. Wong, Rebecca L. Skalsky, Jeremy Smedley, Michael K. Axthelm, Jeffrey D. Lifson, Jacob D. Estes, Paul T. Edlefsen, Louis J. Picker, Cheryl M. Ainslie Cameron, Timothy J. Henrich and Afam A. Okoye.

Corresponding author: Afam A. Okoye, Ph.D.
Vaccine and Gene Therapy Institute
Oregon Health & Science University
505 NW 185th Ave., Beaverton, OR 97006
Phone: (503) 418-2752
FAX: (503) 418-2719
Email: okoyea@ohsu.edu

Supplemental Methods

Animals. A total of 14 purpose-bred male RMs (*M. mulatta*) of Indian genetic background were used for these experiments. These RMs were specific pathogen-free as defined by being free of cercopithecine herpesvirus 1, D-type simian retrovirus, simian T-lymphotropic virus type 1, rhesus rhadinovirus, and *Mycobacterium tuberculosis*. MHC-1 genotyping for common *Mamu* alleles such as *Mamu*-A*01/-A*02 and *Mamu*-B*08/-B*17 was performed by sequence-specific priming PCR, essentially as described (1). RMs (3-7 years of age) were i.v, inoculated with 200 infectious units of the SIVmac239M viral stock (2) and placed on ART starting 12 dpi. ART consisted of subcutaneous injection of 5.1 mg kg^{-1} d^{-1} tenofovir disoproxil, 40 mg kg^{-1} d^{-1} emtricitabine and 2.5 mg kg^{-1} d^{-1} dolutegravir in a solution containing 15% (v/v) kleptose at pH 4.2, as previously described (3). In addition, RMs received twice daily intramuscular injections of rapamycin (LC Laboratories; R-5000) (n=7) or vehicle control [10% (v/v) N,N-Dimethylacetamide (Sigma-Aldrich; 271012), 10% (v/v) Tween®80 (Sigma-Aldrich; P6474) and 80% (v/v) Polyethylene Glycol 400 (Sigma-Aldrich, 91893) (n=7) at 0.02 mg/kg, starting 231 dpi. Anti-CD3LALA (SP34R1LALA) mAb (Cat# PR-2422, RRID:AB_2894696), or IgG isotype control mAb (Cat# PR-1117, RRID:AB_2716330) provided by the Nonhuman Primate Reagent Resource were administered intravenously at 0.5 mg/kg on 476 and 497 dpi. ART and rapamycin were both stopped at 543 dpi.

Rapamycin drug levels. Blood rapamycin (sirolimus) levels were tested using the Abbot Architect Chemiluminescent Immunoassay as previously reported (4). This automated electrochemiluminescence method has been shown to have comparable agreement and precision with liquid chromatography-Mass Spectroscopy methods (5). Testing was performed on EDTA whole blood at room temperature collected prior to animal dosing (*i.e.*, trough value). The therapeutic goal trough value was 10 to 15 ng/mL, representing the higher end of desired trough values used for organ rejection and graft-versus-host prophylaxis in humans (5-15 ng/mL).

Serum chemistry. Serum chemistry analysis was performed by the Oregon National Primate Research Center's Division of Comparative Medicine, Department of Clinical Pathology using the Horiba ABX Petra 400 Clinical Chemistry system (Kyoto, Japan), following protocols supplied by the manufacturer.

LPS, D-dimer, and sCD14 detection assays. Plasma LPS, D-dimer, and sCD14 concentrations were quantified by ELISA in the Endocrine Technologies Core at the Oregon National Primate Research Center. LPS was analyzed following the manufacturer's instructions (Abbexa, Cambridge, United Kingdom). The assay range was 12.35 – 1000 ng/ml. Intra-assay CV was 9.6% and inter-assay CV was 18.1% (n=4 assays).

Plasma samples were diluted 1:10,000 and analyzed for D-dimer following the manufacturer's instructions (Thermo Fisher Scientific, Waltham, MA, USA). The assay range was 0.082 – 60 pg/ml. Intra-assay CV was 4.0% and inter-assay CV was 13.7% (n=4 assays). Soluble CD14 (sCD14) concentrations were determined following manufacturer's instructions (R&D Systems, Minneapolis, MN, USA) after samples were diluted 1:100. The assay range was 250-16,000 pg/ml. Intra-assay CV was 5.1% and inter-assay CV was 6.2% (n=3 assays).

Viruses. The SIVmac239M challenge stock used in this experiment was produced in transfected HEK-239T cells and the stock infectivity titer was determined using TZM-bl cells as previously described (2).

SIV viral detection assays. Plasma SIV RNA levels were determined using a gag-targeted quantitative real time/digital RT-PCR format assay, essentially as previously described, with 6 replicate reactions analyzed per extracted sample for assay threshold of 15 SIV RNA copies/ml (6). Ultrasensitive determinations of plasma SIV RNA were measured by concentrating virus from larger volumes of plasma by centrifugation. For ultrasensitive measurements, typically, 1.7 ml of plasma were centrifuged in a refrigerated microfuge (21,000 x g, 1 hr, 4° C) and nucleic acid was extracted from pellets as described (7) and quantitative RT PCR was performed with 12 reactions per extracted sample. Samples that did not yield any positive results across the replicate reactions were reported as a value of "less than" the value that would apply for one positive reaction out of 12 (6). As performed, the ultrasensitive assay provided a threshold sensitivity of 1 copy/ml plasma for a 1.7 ml sample. Quantitative assessment of SIV DNA and RNA in cells and tissues was performed using gag targeted nested quantitative hybrid real-time/digital RT-PCR and PCR assays, as previously described (6, 8). SIV RNA or DNA copy numbers were normalized based on quantitation of a single copy rhesus genomic DNA sequence from the CCR5 locus from the same specimen to allow normalization of SIV RNA or DNA copy numbers per 10^6 diploid genome cell equivalents, as described (9). Ten replicate reactions were performed with aliquots of extracted DNA or RNA from each sample, with two additional spiked internal control reactions performed with each sample to assess potential reaction inhibition. Samples that did not yield any positive results across the replicate reactions were reported as a value of "less than" the value that would apply for one positive reaction out of 10. Threshold sensitivities for individual specimens varied as a function of the number of cells or amount of tissue available and analyzed.

LCV and RRV viral detection assays. Total DNA was purified from whole blood and resuspended in nuclease-free water. One hundred nanograms of total DNA were analyzed in duplicate by qPCR using primers and Taqman probe specific to the rLCV IR1 repeat region or to the RRV ORF3, as previously described (10).

Immunofluorescence and quantitative image analysis. To phenotype Glut1-expressing cells, fluorescent microscopy was performed on lymph node biopsies that were fixed in 4% paraformaldehyde and paraffin embedded; 5 µm sections were deparaffinized in xylene and rehydrated through a series of graded ethanol to distilled water. Heat-induced epitope retrieval (HIER) was performed with citraconic anhydride (0.01% + 0.05% Tween-20) in a Biocare NxGen Decloaking Chamber at 110°C for 15 min. Slides were stained with anti-Glut1 (SPM498: Abcam, ab40084; 1:500 for 1 h), Mouse Polink-1 HRP (GBI Labs, D12-110; 20 min.), and developed with Alexa Fluor 647 tyramide (Invitrogen, B40958; 1:500 for 10 min.). Endogenous peroxidase was blocked with 1.5% H_2O_2 in TBS-T for 5 min. following Glut1 antibody incubation. Antibody stripping was performed by heating slides at 95°C for 10 min. in Target Retrieval (Advanced Cell Diagnostics, 322100) following Glut1 development. Slides were further stained with a cocktail of CD3 (LN10: Biocare, ACI3152C; 1:100 for 1 h) and CD20 (Invitrogen, PA1-9024; 1:800 for 1 h), followed by a cocktail of donkey anti-mouse DyLight 755 (Invitrogen, SA5-10171; 1:500 for 1 h) and donkey anti-goat Alexa Fluor 568 (Invitrogen, A11057; 1:500 for 1 h). All staining was performed at room temperature, and slides were counter-stained with 4',6-diamidino-2-phenylindole (DAPI; Invitrogen, D1306; 0.5µg/ml for 10 min.), mounted in Prolong Gold Antifade Mountant (Invitrogen, P36930), and scanned at 20x magnification on an AxioScan.z1 (Zeiss). Quantitative image analysis was performed using HALO software (Indica Labs, v3.2.1851.393) on at least two lymph node sections from each animal and timepoint. The percentage of $Glut1^+$ T cells was determined with the HighPlex FL v4.0.4 module using positive selection for CD3 and negative selection on green/red autofluorescence (to exclude RBCs, which strongly express Glut1). Each sample was manually double-checked to ensure accurate quantification.

Sequencing and qRT-PCR analysis of circulating miRNAs. Small RNAs were isolated from 200 ul archived plasma on days -14 and 42 post-rapamycin using the miRNeasy mini kit (Qiagen) and eluted into nuclease-free water. miRNA sequencing libraries were prepared using the NEBNext Small RNA Library Prep Set for Illumina (NEB) according to the manufacturer's protocol. Libraries were multiplexed and sequenced on the Illumina HiSeq2000 at the OHSU Massively Parallel Sequencing Shared Resource. Sequencing reads were obtained in FASTQ format and pre-processed to clip adapter sequences and collapse duplicate reads via the FASTX-Toolkit (http://hannonlab.cshl.edu/fastx_toolkit). Reads were aligned using

bowtie (-v 2 -m 25 -l 17 –best --strata) to the rhesus macaque genome, mmul8 (GCA_000772875.3). Mapped reads were subsequently analyzed by miRDeep2 (11) to obtain read counts per miRNA. Determination of differentially expressed miRNAs was performed using the Bioconductor package, edgeR (12). Library sizes were normalized by using the edgeR default weighted trimmed mean of *M* values (TMM) method and pairwise comparisons were performed between two groups (i.e., rapamycin versus vehicle). miRNAs were considered to be significantly differentially expressed between groups when the false discovery rate (FDR) p-value was < 0.05. To analyze miRNA expression by qRT-PCR, six microliters of RNA were used in a 40 ul reverse transcriptase (RT) reaction with pooled stem-loop RT primers specific for miR-16, miR-18a, miR-21, miR-23a-3p, miR-26a, miR-28, miR-103-5p, and miR-155. qPCR reactions were set up in duplicate for each sample using 3 ul of the RT reaction and the corresponding miRNA Taqman probe. All miRNA qRT-PCR values were normalized to miR-16 and set relative to the control cohort at d-14. Raw sequence data files are accessible through NCBI SRA (short read archive), BioProject ID PRJNA772267.

RNA-Seq and bioinformatic analysis. RNA was purified from whole blood collected on days 84 and 181 post-rapamycin or vehicle control in Paxgene vacutainers using PreAnalytiX Paxgene Blood RNA kit (Qiagen), followed by RNASeq library generation using Illumina TruSeq Stranded Total RNA – Globin kit (Illumina). Paired-end sequencing reactions were run on an Illumina NextSeq 550 High-output platform (30M total reads per sample). Raw demultiplexed FASTQ paired end read files were trimmed of adapters and filtered using the program skewer (13) to discard those with an average phred quality score of less than 30 or a length of less than 36. Trimmed reads were then aligned using the HISAT2 (14) aligner to the Macaca mulatta NCBI reference genome assembly version Mmul_8 and sorted using SAMtools (15). Aligned reads were counted and assigned to gene meta-features using the program featureCounts (16) as part of the Subread package. These count files were imported into the R programming language and were assessed for quality control, normalized and analyzed using an in-house pipeline utilizing the limma-trend method (17) for differential gene expression testing and the GSVA (18) library for gene set sample enrichment. Final differential gene expression lists were filtered to remove non-coding RNAs as well as LOC features. RNA-Seq data have been deposited in the NCBI's Gene Expression Omnibus database (GEO Accession # Pending).

Immunophenotyping. To determine the phenotype of lymphocyte populations, whole blood or mononuclear cell preparations from LN and BM were stained for flow-cytometric analysis as previously described (19-21). Polychromatic (8-14 parameter) flow-cytometric analysis was performed on an LSR II

BD instrument using Pacific blue, BUV395, BUV495, BUV737, BUV805, BV421, BV510, BV570, BV605, BV650, BV711, BV786, FITC, PE, PE-Texas red (PE-CF594), PE-Cy7, PerCP-Cy5.5, APC, APC-Cy7, and Alexa 700 as the available fluorescent parameters. Instrument setup and data acquisition procedures were performed as previously described (19-21). List mode multiparameter data files were analyzed using the FlowJo software program (Tree Star). Criteria for delineating T_N and T_M subsets and for setting positive (+) versus negative (−) markers for CCR5 and Ki-67 expression have been previously described in detail (19-21). In brief, T_N constitute a uniform cluster of cells with a $CD28^{moderate}$, $CCR7^+$, $CCR5^-$, $CD95^{low}$ phenotype, which is clearly distinguishable from the phenotypically diverse memory population that is $CD95^{high}$ or displays one or more of the following non-naive phenotypic features: $CD28^-$, $CCR7^-$, $CCR5^+$. The T_{CM}, transitional memory T cell (T_{TrM}), and effector memory T cell (T_{EM}) components of the memory subset in the blood were further delineated based on the following phenotypic criteria: T_{CM} ($CD28^+$, $CCR7^+$, $CCR5^-$), T_{TrM} ($CD28^+$, $CCR7^{+/-}$, $CCR5^+$), and T_{EM} ($CD28^-$, $CCR7^-$, $CCR5^{dim}$). For each subset to be quantified, the percentages of the subset within the overall small lymphocyte and/or small T cell ($CD3^+$ small lymphocyte) populations were determined. For quantification of peripheral blood subsets, absolute small lymphocyte counts were obtained using an AcT5diff cell counter (Beckman Coulter) and, from these values, absolute counts for the relevant subset were calculated based on the subset percentages within the light scatter–defined small lymphocyte population on the flow cytometer. Baseline values were determined as the average of values at days −14, −7, and 0. Absolute counts are indicated as percentage change from baseline with baseline shown as 100%. Changes in proliferative fraction are indicated as the difference in the $\%Ki\text{-}67^+$ ($\Delta\%Ki\text{-}67^+$) measured at the designated time points from baseline (0% = no change). Combinations of fluorochrome-conjugated monoclonal antibodies used for staining included anti-CD3 (SP34-2: BUV395; BD Biosciences, Custom Bulk 624310 and APC-Cy7; BD Biosciences, Custom Bulk 624072, FN18: FITC; Nonhuman Primate Reagent Resource, Cat# PR-3180, RRID:AB_2819276), anti-CD4 (L200: BV786; BD Biosciences, Custom Bulk 624159 and BV510; BD Biosciences, Custom Bulk 624340), anti-CD8α (DK25: Pacific Blue; SK1: BUV737; BD Biosciences, Custom Bulk 624235, BV711; BD Biosciences, Custom Bulk 624148 and BV510; BD Biosciences, Custom Bulk 624144), anti-CD95 (DX2: PE; BioLegend, Custom Bulk 94203 and APC; ebioscience, Custom Bulk 7017-0959-M050 and BUV737; BD Biosciences, Custom Bulk 624231), anti-CD28 (CD28.2: PE-DAZZ; BioLegend, Custom Bulk 93364), anti-CCR5 (3A9: APC; BD Biosciences, Custom Bulk 624076), anti-Ki67 (B56: FITC; BD Biosciences, Custom Bulk 624046 and PE; BD Biosciences, Custom Bulk 624048), anti-CD14 (M5E2: FITC; BioLegend, Custom Bulk 94202), anti-CD16 (3G8: BV650; BD Biosciences, Custom Bulk 93384), anti-HLA-DR (L243: PE-DAZZ; BioLegend, Custom Bulk 93957 and BV510; BioLegend, Custom Bulk 93784), anti-CD20 (2H7: APC-Cy7; BioLegend, Custom Bulk 93924), anti-CCR7 (150503: Biotin; R&D Systems, MAB197 and BV711, BD Biosciences, Custom Bulk 624386), anti-

NKG2A (REA110: APC; Miltenyi, 130-095-212 and PE, Miltenyi, 130-095-212), anti-CD56 (MEM-188: PerCP-Cy5.5, Life Technologies, MHCD5618CS3), anti-CD69 (CH/4: PerCP-Cy5.5; Life Technologies, MHCD6918), anti-PD-1 (eBioJ105: PerCP-Cy5.5; Life Technologies, Custom Bulk CUST00656), anti-CXCR3 (G025H7: BV510; BioLegend, Custom Bulk 94271), anti-CXCR5 (MU5UBEE: PE; Life Technologies, Custom Bulk CUST03534), anti-CD169 (7-239: PE; BioLegend, Custom Bulk 346004) and anti-streptavidin (BV421; BD Biosciences, Custom Bulk 624337 and BV605, BD Biosciences Custom Bulk 624342).

Statistics. Statistical analyses were performed in R 3.6.0 with the package "survival" v3.2-11. We used Spearman rank transformation when evaluating correlations. We used Wilcoxon rank-sum (WRS) tests for all analyses comparing values across treatment groups. Point values were transformed to the log10 scale where indicated. For analyses involving multiple timepoints, we calculated the area under the curve (AUC), peak value, or number of 'blips' (observations above a pre-defined threshold) for each RM and analyzed the resulting values in a similar fashion to single-timepoint data. Time-to-event data were described with Kaplan-Meier estimates, and compared between groups using the Kruskal-Wallis test. All tests were conducted with two-sided null hypotheses at significance level $P \leq 0.05$, except for experiments on the effect of anti-CD3LALA (Figure 8A-B), for which we used two separate one-sided tests with significance level $P \leq 0.025$. While we present primarily unadjusted p-values in accordance with our pre-specified plan, and consistent with our usual practice, we value and encourage consideration of the impacts of multiple testing. For post-hoc multiplicity adjustment over any set of tests, we provide all unadjusted p-values in **Supplementary Table 3**. The datasets generated and/or analyzed during the current study, as well as computer code used to perform statistical analysis are available in **Supplementary Data and Code**.

Sample size and treatment assignment. Sample size was determined by logistical and resource considerations. Treatment assignments (rapamycin vs. vehicle control treatment) were conducted after 231 dpi by assigning alternating treatments arbitrarily (but not randomly) and without revision. No blinding was possible due to the constraints of working with RM. No RMs were excluded from any analysis in this study, except the following; (1) Analysis of differential miRNA expression (Figure 2 and Supplementary Figure 3) and correlation between miRNA expression and $CD4^+$ T_M proliferation (Supplementary Figure 4); miRNA sequencing libraries for one animal in the rapamycin group failed initial QC steps (low read depth) and were therefore not included in the final analysis of these data. (2) Analysis of post-ART SIV infection dynamics (Figure 9) and post-ART $CD8^+$ T_M, B cell and NK cell proliferation (Supplementary Figure 15);

as one animal in the rapamycin group was lost from study prior to ART withdrawal due to non-study related health complications and therefore not included in the final analysis of these data.

References

1. Loffredo JT, Maxwell J, Qi Y, Glidden CE, Borchardt GJ, Soma T, et al. Mamu-B*08-positive macaques control simian immunodeficiency virus replication. *J Virol.* 2007;81(16):8827-32.
2. Fennessey CM, Pinkevych M, Immonen TT, Reynaldi A, Venturi V, Nadella P, et al. Genetically-barcoded SIV facilitates enumeration of rebound variants and estimation of reactivation rates in nonhuman primates following interruption of suppressive antiretroviral therapy. *PLoS Pathog.* 2017;13(5):e1006359.
3. Del Prete GQ, Smedley J, Macallister R, Jones GS, Li B, Hattersley J, et al. Short Communication: Comparative Evaluation of Coformulated Injectable Combination Antiretroviral Therapy Regimens in Simian Immunodeficiency Virus-Infected Rhesus Macaques. *AIDS Res Hum Retroviruses.* 2016;32(2):163-8.
4. Schmid RW, Lotz J, Schweigert R, Lackner K, Aimo G, Friese J, et al. Multi-site analytical evaluation of a chemiluminescent magnetic microparticle immunoassay (CMIA) for sirolimus on the Abbott ARCHITECT analyzer. *Clin Biochem.* 2009;42(15):1543-8.
5. Stove V, Ramos PA, Wallemacq P, Vogeser M, Schuetzenmeister A, Schmiedel C, et al. Measurement of sirolimus concentrations in human blood using an automated electrochemiluminescence immunoassay (ECLIA): a multicenter evaluation. *Clin Chem Lab Med.* 2018;56(5):764-75.
6. Hansen SG, Piatak M, Ventura AB, Hughes CM, Gilbride RM, Ford JC, et al. Addendum: Immune clearance of highly pathogenic SIV infection. *Nature.* 2017;547(7661):123-4.
7. Li H, Wang S, Kong R, Ding W, Lee FH, Parker Z, et al. Envelope residue 375 substitutions in simian-human immunodeficiency viruses enhance CD4 binding and replication in rhesus macaques. *Proc Natl Acad Sci U S A.* 2016;113(24):E3413-22.
8. Hansen SG, Piatak M, Jr., Ventura AB, Hughes CM, Gilbride RM, Ford JC, et al. Immune clearance of highly pathogenic SIV infection. *Nature.* 2013;502(7469):100-4.
9. Venneti S, Bonneh-Barkay D, Lopresti BJ, Bissel SJ, Wang G, Mathis CA, et al. Longitudinal in vivo positron emission tomography imaging of infected and activated brain macrophages in a macaque model of human immunodeficiency virus encephalitis correlates with central and peripheral markers of encephalitis and areas of synaptic degeneration. *Am J Pathol.* 2008;172(6):1603-16.
10. Okoye AA, DeGottardi MQ, Fukazawa Y, Vaidya M, Abana CO, Konfe AL, et al. Role of IL-15 Signaling in the Pathogenesis of Simian Immunodeficiency Virus Infection in Rhesus Macaques. *J Immunol.* 2019;203(11):2928-43.
11. Friedlander MR, Mackowiak SD, Li N, Chen W, and Rajewsky N. miRDeep2 accurately identifies known and hundreds of novel microRNA genes in seven animal clades. *Nucleic Acids Res.* 2012;40(1):37-52.
12. Robinson MD, McCarthy DJ, and Smyth GK. edgeR: a Bioconductor package for differential expression analysis of digital gene expression data. *Bioinformatics.* 2010;26(1):139-40.
13. Jiang H, Lei R, Ding SW, and Zhu S. Skewer: a fast and accurate adapter trimmer for next-generation sequencing paired-end reads. *BMC Bioinformatics.* 2014;15:182.
14. Kim D, Langmead B, and Salzberg SL. HISAT: a fast spliced aligner with low memory requirements. *Nat Methods.* 2015;12(4):357-60.

15. Yang J, Ding X, Sun X, Tsang SY, and Xue H. SAMSVM: A tool for misalignment filtration of SAM-format sequences with support vector machine. *J Bioinform Comput Biol.* 2015;13(6):1550025.
16. Liao Y, Smyth GK, and Shi W. featureCounts: an efficient general purpose program for assigning sequence reads to genomic features. *Bioinformatics.* 2014;30(7):923-30.
17. Ritchie ME, Phipson B, Wu D, Hu Y, Law CW, Shi W, et al. limma powers differential expression analyses for RNA-sequencing and microarray studies. *Nucleic Acids Res.* 2015;43(7):e47.
18. Hanzelmann S, Castelo R, and Guinney J. GSVA: gene set variation analysis for microarray and RNA-seq data. *BMC Bioinformatics.* 2013;14:7.
19. Okoye A, Meier-Schellersheim M, Brenchley JM, Hagen SI, Walker JM, Rohankhedkar M, et al. Progressive CD4+ central memory T cell decline results in CD4+ effector memory insufficiency and overt disease in chronic SIV infection. *J Exp Med.* 2007;204(9):2171-85.
20. Okoye A, Park H, Rohankhedkar M, Coyne-Johnson L, Lum R, Walker JM, et al. Profound CD4+/CCR5+ T cell expansion is induced by CD8+ lymphocyte depletion but does not account for accelerated SIV pathogenesis. *J Exp Med.* 2009;206(7):1575-88.
21. Okoye AA, Rohankhedkar M, Abana C, Pattenn A, Reyes M, Pexton C, et al. Naive T cells are dispensable for memory CD4+ T cell homeostasis in progressive simian immunodeficiency virus infection. *J Exp Med.* 2012;209(4):641-51.

Supplemental Figures and Table

Animal ID	Treatment group	Sex	Age at time of SIV infection (years)	MHC	Weight (kg)
C1*	Rapamycin	Male	6	A*01	7.4
C2	Rapamycin	Male	6	A*01	6.6
C3	Rapamycin	Male	5	A*01	8.4
C4	Rapamycin	Male	4		7.2
C5	Rapamycin	Male	7	A*01	8.6
C6	Rapamycin	Male	3		4.8
C7	Rapamycin	Male	4		3.8
D1	Control	Male	3		6.2
D2	Control	Male	4		5.6
D3	Control	Male	4	A*01	6.2
D4	Control	Male	4	A*01	5.6
D5	Control	Male	5	A*01	6.6
D6	Control	Male	3		4.4
D7	Control	Male	3		4.6

* Lost from study prior to ART release

Supplementary Table 1: Characteristics of rapamycin-treated and control RM. The table shows the sex, age, MHC-1 alleles and weight of RMs at the time of SIV infection.

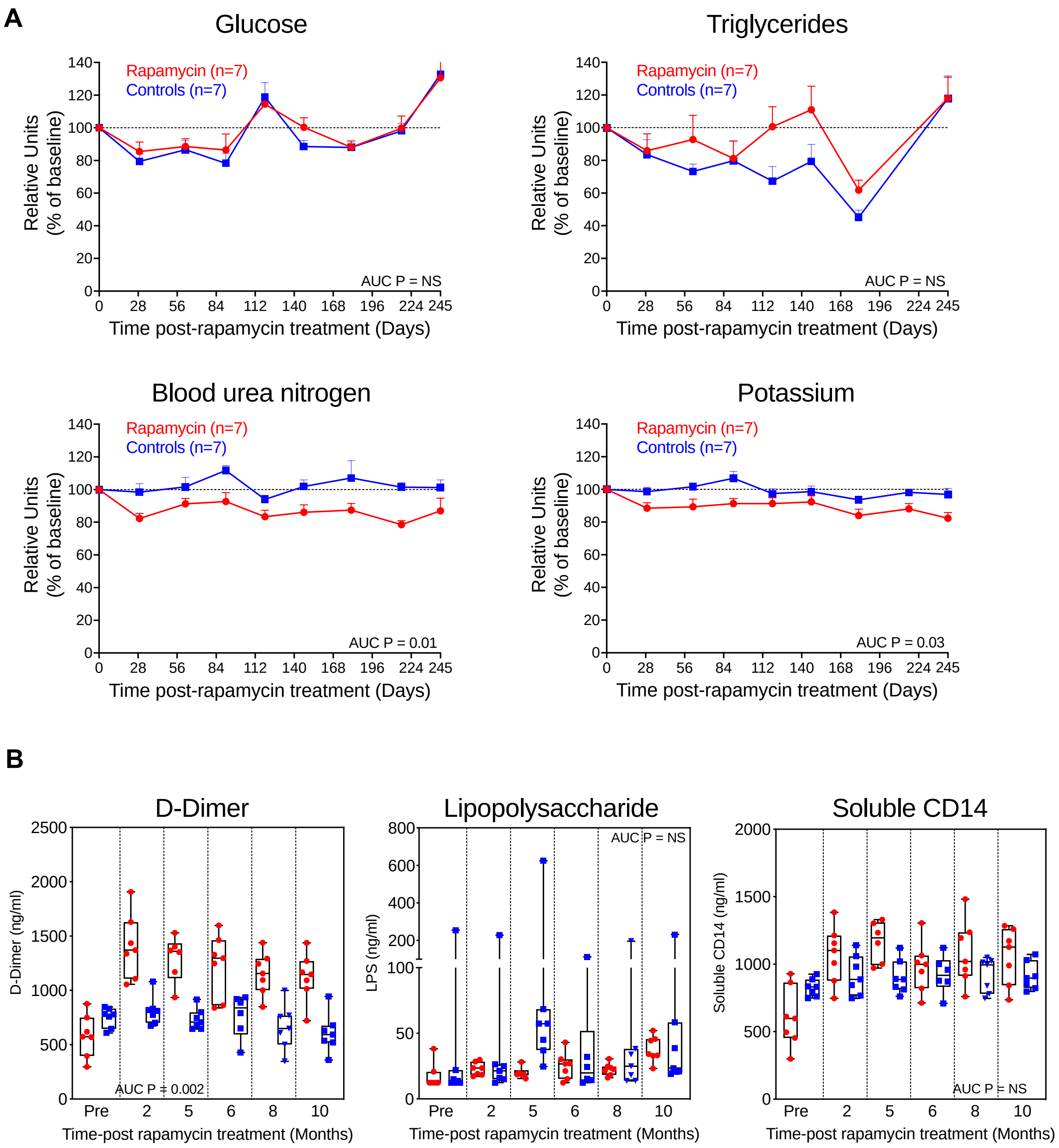

Supplementary Figure 1: Effect of rapamycin treatment on blood chemistry. (**A**) Mean (+SEM) change from baselines of glucose, triglycerides, blood urea nitrogen and potassium in blood of rapamycin-treated RMs (n = 7) vs. vehicle controls (n = 7). (**B**) Quantification of D-dimer, lipopolysaccharide (LPS) and soluble CD14 (sCD14) in plasma of rapamycin-treated RMs (n = 7) vs. vehicle controls (n = 7). The WRS test was used to determine the significance of differences in AUC between the two treatment groups (p-values ≤ 0.05 are shown). Boxplots show jittered points and a box from 1st to 3rd quartiles (IQR) and a line at the median, with whiskers extending to the farthest data point within 1.5 × IQR above and below the box.

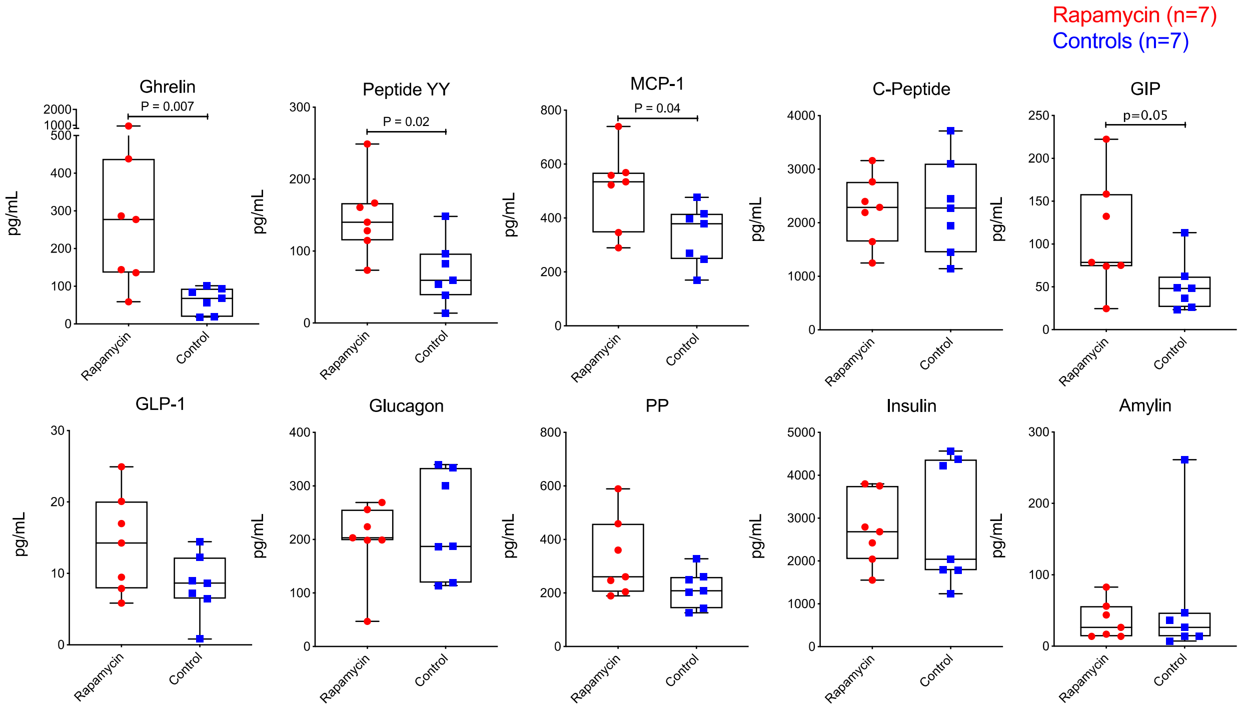

Supplementary Figure 2: Effect of rapamycin treatment on metabolic hormones. Quantification of metabolic hormone levels in plasma after 34 weeks of treatment with rapamycin (red, n = 7) or vehicle control (blue, n = 7). Each data point represents a single determination from an individual RM. The WRS test was used to determine the significance of differences between the rapamycin or vehicle control treatment groups (p-values $\leq$ 0.05 are shown). Pancreatic polypeptide (PP), monocyte chemoattractant protein-1 (MCP-1), gastric inhibitory polypeptide (GIP), glucagon-like peptide-1 (GLP-1). Boxplots show jittered points and a box from 1st to 3rd quartiles (IQR) and a line at the median, with whiskers extending to the farthest data point within 1.5 × IQR above and below the box.

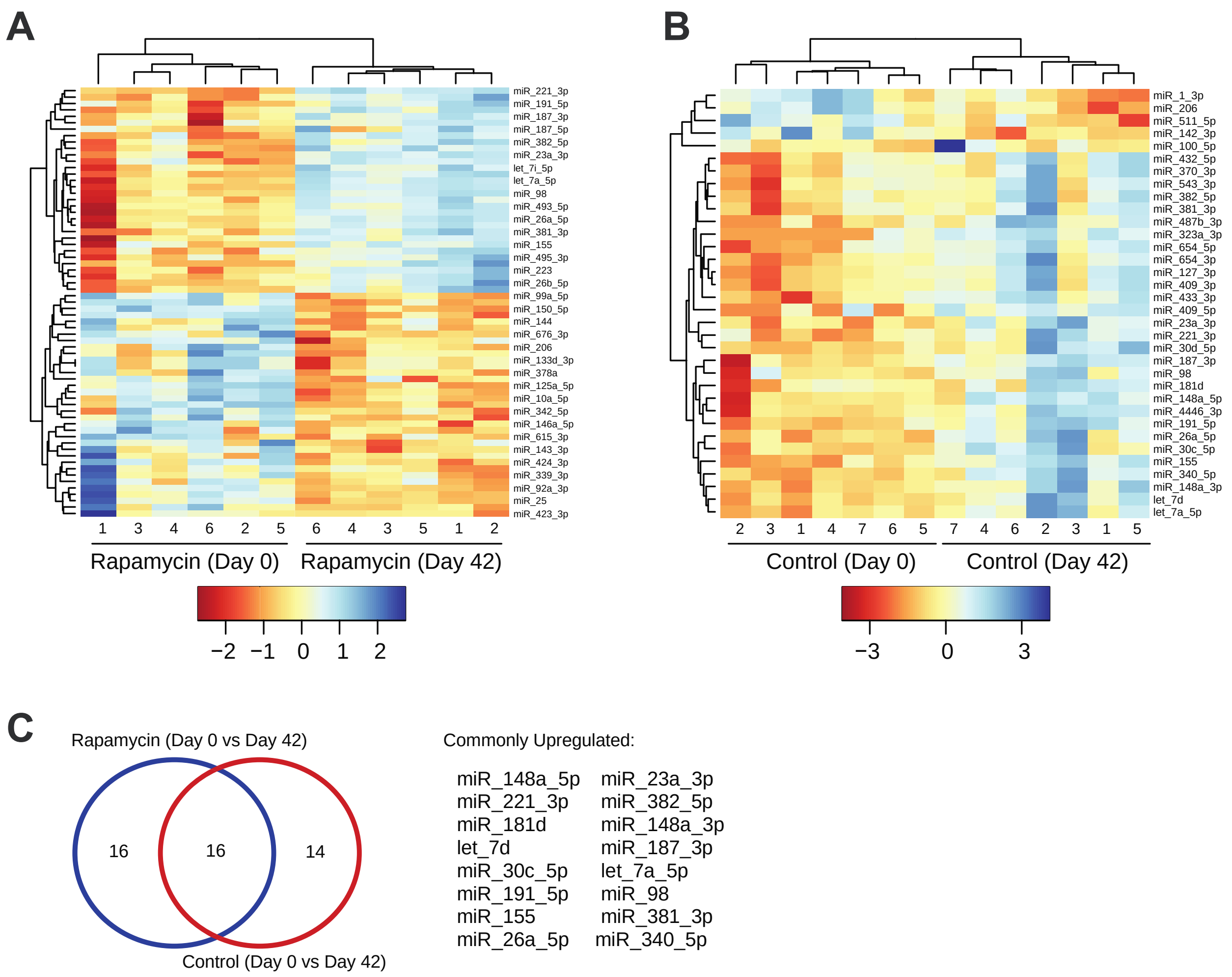

Supplementary Figure 3: Effect of rapamycin treatment on miRNAs in plasma. (**A**) Heatmap of significant differentially expressed miRNAs in the plasma of rapamycin-treated RM (n=6) between day 0- and 42-days post-treatment. For these analyses n=13; miRNA sequencing libraries for one animal in the rapamycin group failed initial QC steps (low read depth) and were therefore not included in the final analysis. (**B**) Heatmap of significant differentially expressed miRNAs in the plasma of vehicle control-treated RM (n=7) between day 0- and 42-days post-treatment. (**C**) Venn diagram showing miRNAs that were commonly upregulated in both treatment groups after 42 days of treatment. One rapamycin treated RM was removed from all analyses due to RNA quality that did not meet standards.

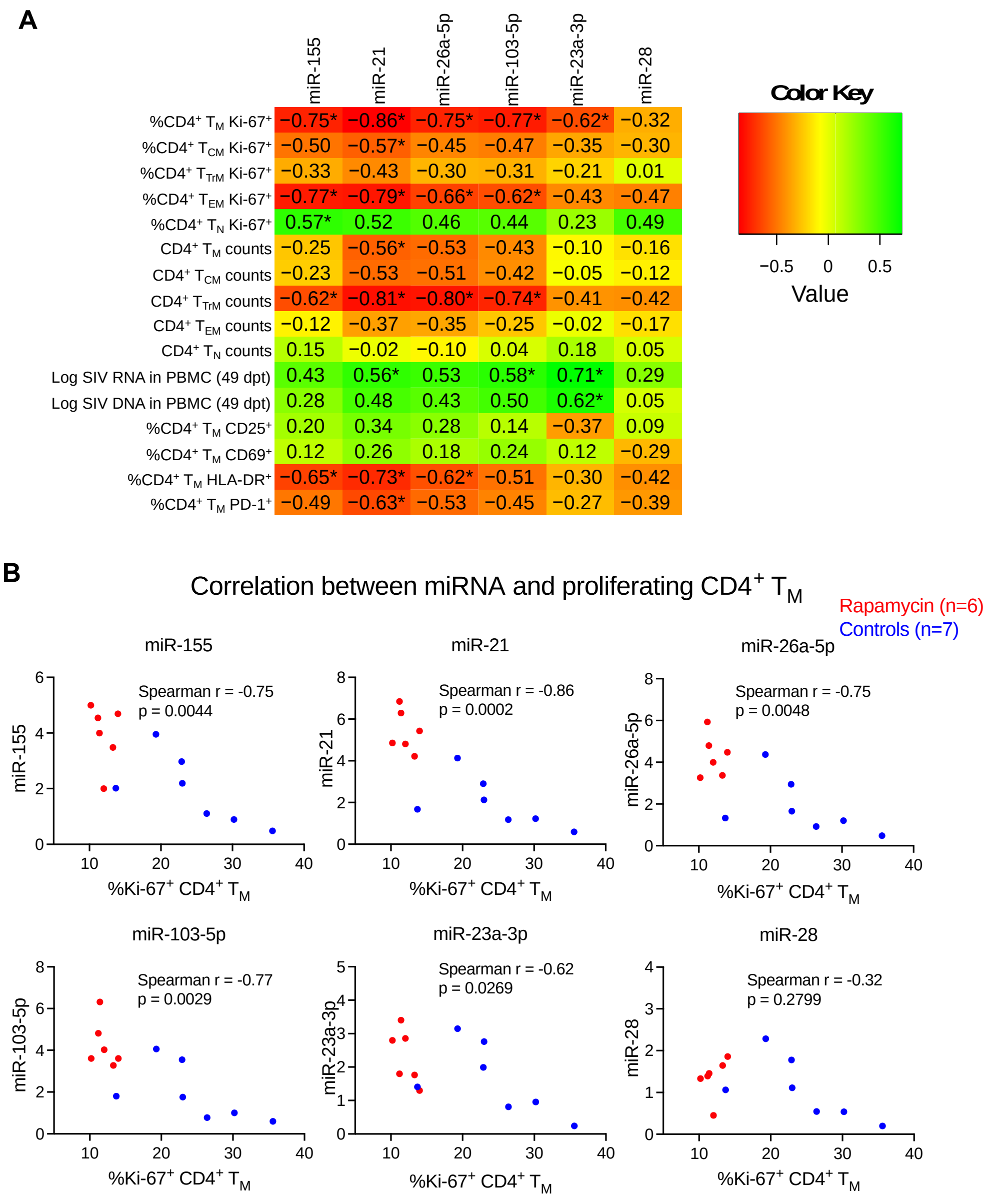

Supplementary Figure 4: Correlation between miRNA expression and CD4$^+$ T$_M$ proliferation. (**A**) Heatmap showing correlations between miR-155, miR-21, miR-26a, miR-103-5p, miR-23a-3p and miR-28 expression levels in plasma versus % Ki-67 and absolute counts of CD4$^+$ total memory (T$_M$), central memory (T$_{CM}$), transitional memory (T$_{TrM}$), effector memory (T$_{EM}$), naïve (T$_N$) T cells, and % CD25, % HLA-DR and % PD-1 on CD4$^+$ T$_M$ at 42 days of rapamycin treatment, and levels of cell-associated SIV RNA and DNA in PBMC at 49 days post-rapamycin treatment (dpt). For these analyses n=13; miRNA sequencing libraries for one animal in the rapamycin group failed initial QC steps (low read depth) and were therefore not included in the final analysis. Unadjusted p-values of Spearman correlation tests < 0.05 are indicated by * after the Spearman correlation value shown on the heatmap. (**B**) Scatterplots of miR-155, miR-21, miR-26a, miR-103-5p, miR-23a-3p and miR-28 expression levels in plasma vs. % Ki-67$^+$ CD4$^+$ T$_M$ in blood at day 42 post-rapamycin treatment. Spearman rank correlation coefficient r with unadjusted p values testing association between paired samples are shown. Rapamycin-treated RM are indicated as treated in red (n = 6) while vehicle control RM are indicated as untreated in blue (n = 7).

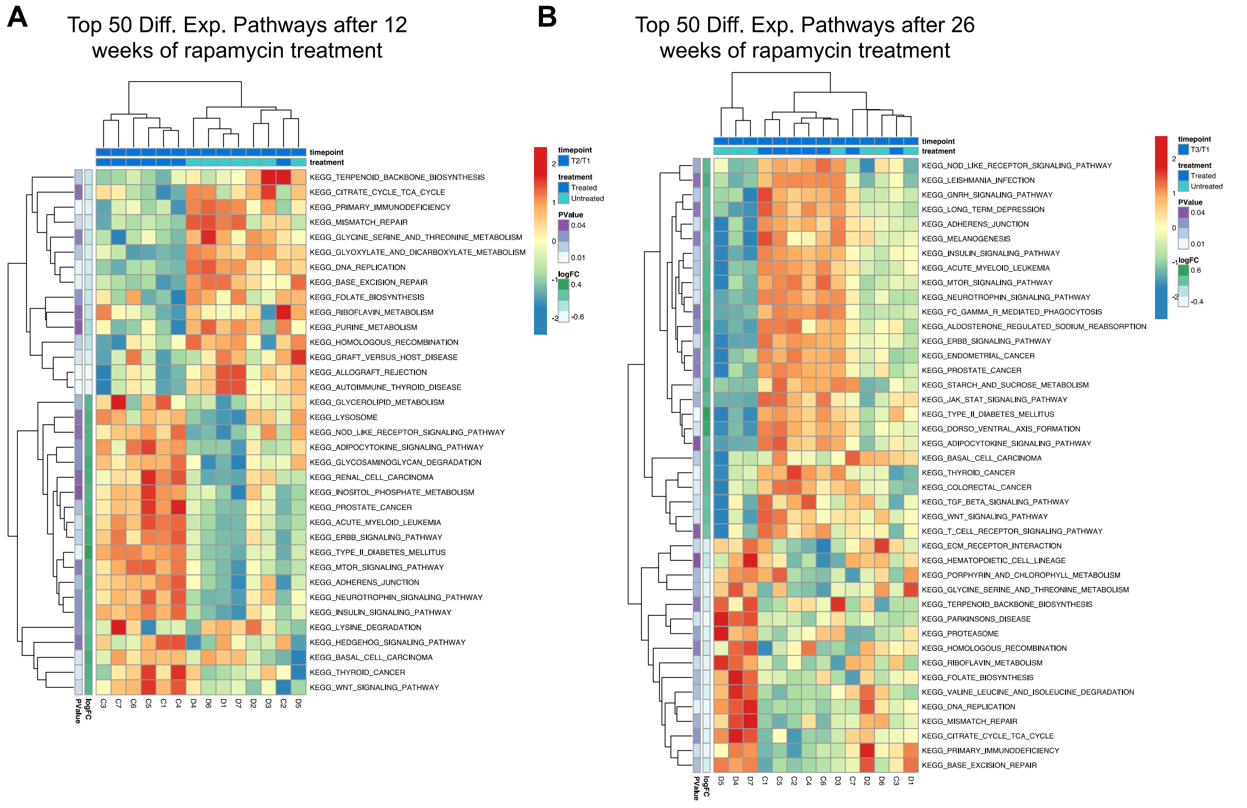

Supplementary Figure 5: Effect of rapamycin treatment on gene expression pathways. (**A**) Heatmaps of differentially enriched pathways following 12 weeks of rapamycin. (**B**) Heatmaps of differentially enriched pathways following 26 weeks of rapamycin. Rapamycin-treated RM are indicated as treated in dark blue (n = 7) while vehicle control RM are indicated as untreated in light blue (n = 7).

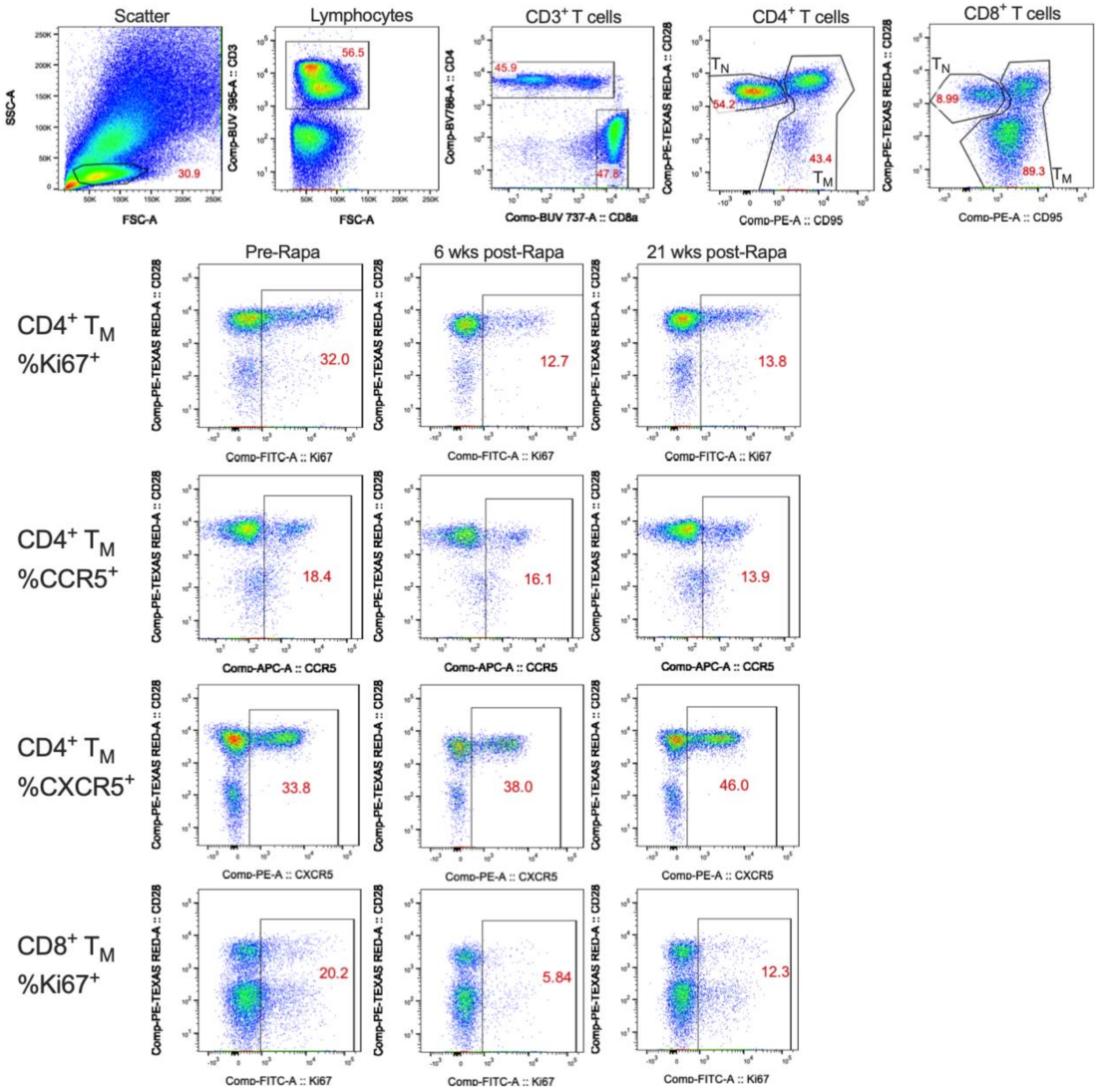

Supplementary Figure 6: Effect of rapamycin treatment on T cells in blood. Representative gating strategy for the flow cytometric analysis of Ki-67, CCR5, CXCR5 expression on CD4$^+$ T$_M$ and Ki-67 on CD8$^+$ T$_M$ in peripheral blood of an SIV-infected RM on ART at 0-, 6- and 21-weeks post-rapamycin treatment. Lymphocytes were gated based on SSC and FSC and then T cells were gated as CD3$^+$. Next, we gated CD4$^+$ and CD8$^+$ T cells on total CD3 followed by CD28 and CD95 to delineate naïve (CD28$^+$ CD95$^-$) and total memory (CD28$^{+/-}$ CD95$^+$).

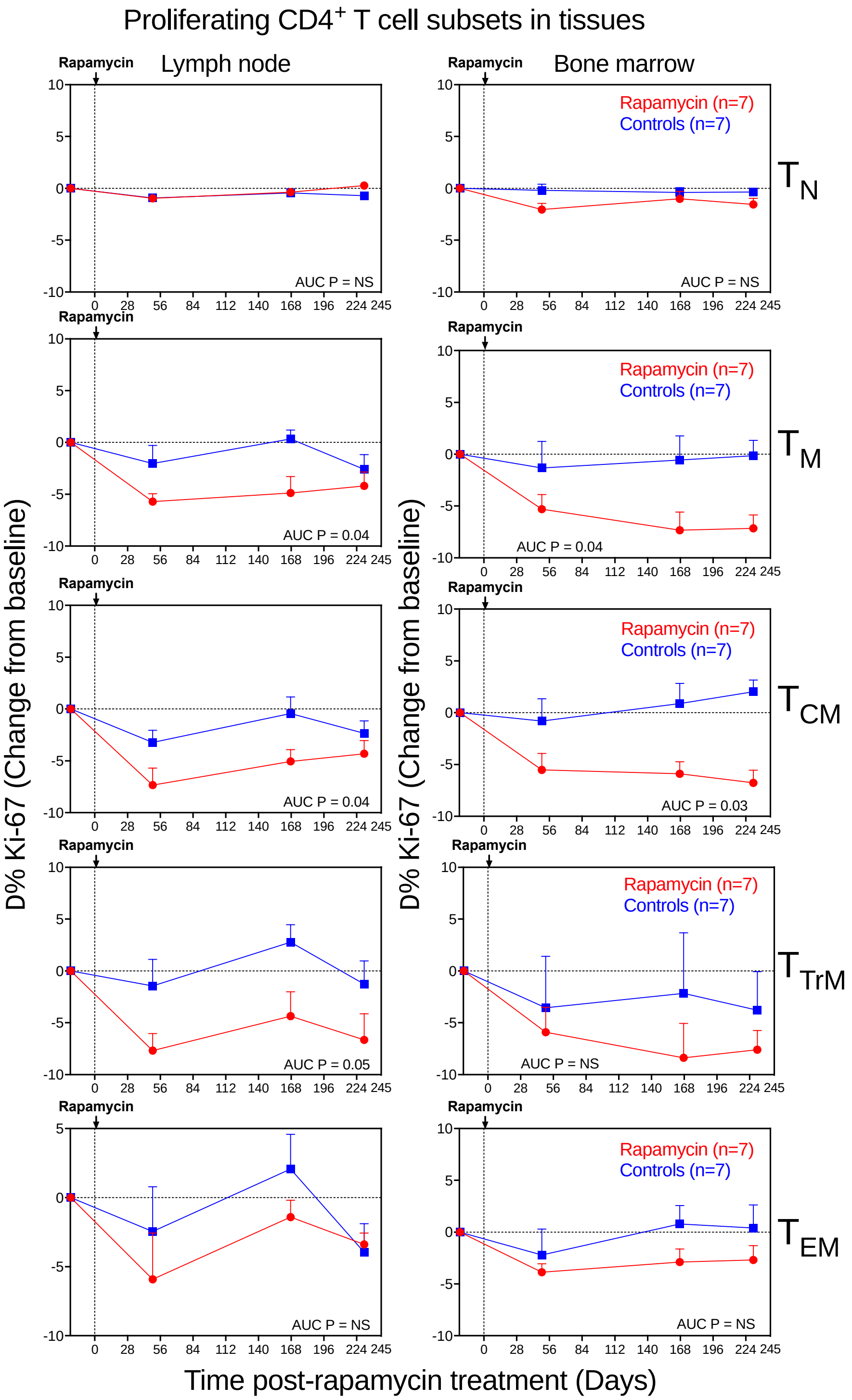

Supplementary Figure 7: Effect of rapamycin treatment on CD4$^+$ T cell subset dynamics in tissues. Change in the proliferative fraction of CD4$^+$ T subsets, including naïve (T_N) and total memory (T_M), central memory (T_{CM}), transitional memory (T_{TrM}), and effector memory (T_{EM}) in lymph node (left panels) and bone marrow (right panels) following rapamycin (n = 7) vs. vehicle control (n = 7) treatment. Results are shown as mean (+ SEM) change from baseline of % Ki-67. The WRS test was used to determine the significance of differences in AUC between the two treatment groups (p-values ≤ 0.05 are shown).

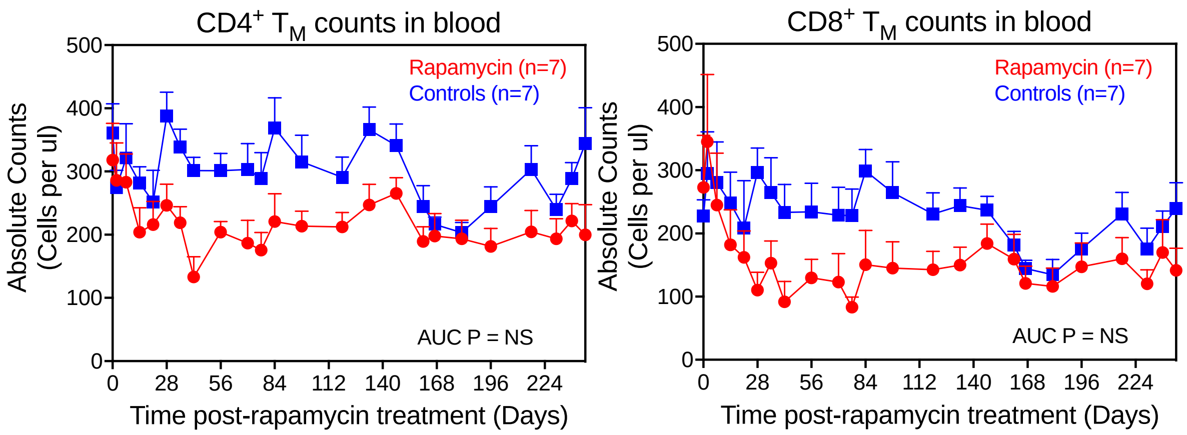

Supplementary Figure 8: Effect of rapamycin treatment on T cell counts in blood. Mean (+ SEM) absolute counts of CD4$^+$ T$_M$ (left panel) and CD8$^+$ T$_M$ (right panel) following rapamycin (n = 7) vs. vehicle control (n = 7) treatment. The WRS test was used to determine the significance of differences in AUC between the two treatment groups.

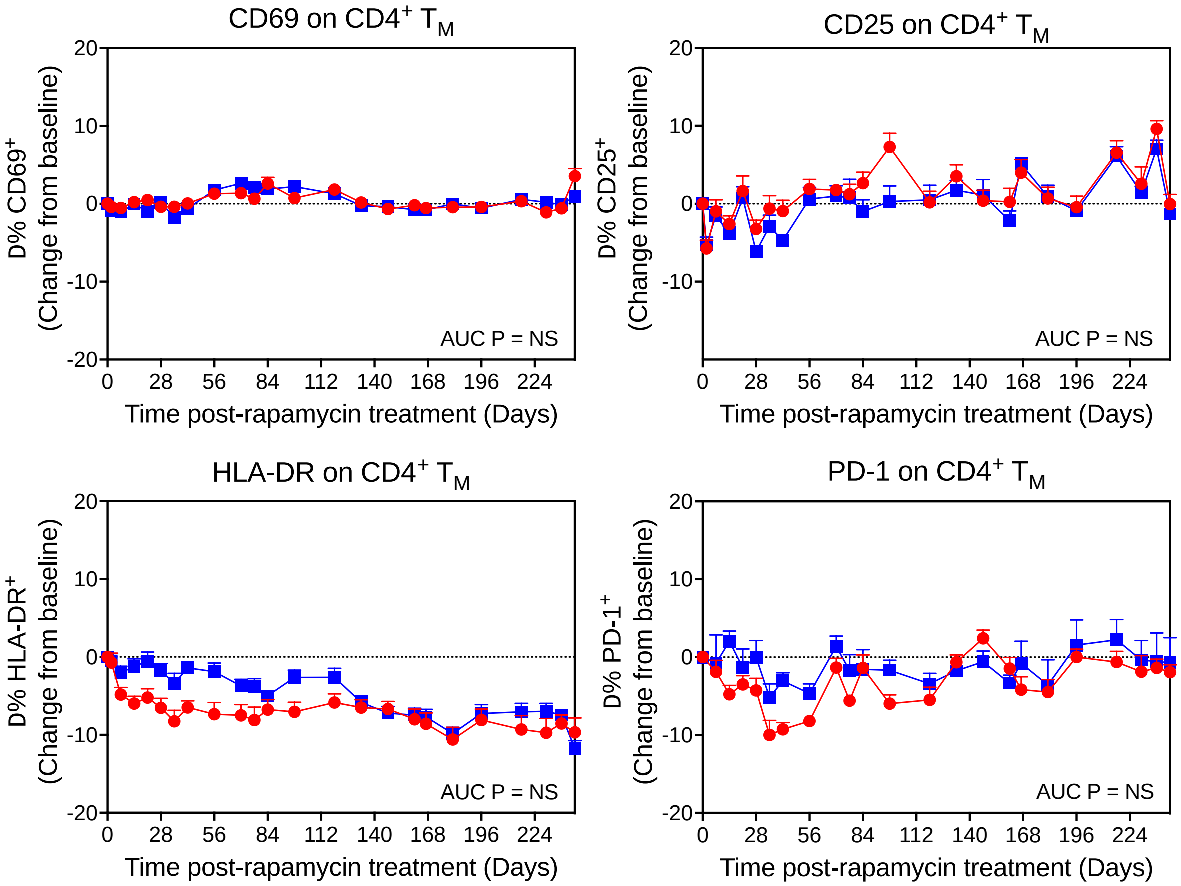

Supplementary Figure 9: Effect of rapamycin treatment on markers of $CD4^+$ T_M activation and exhaustion. Mean (+ SEM) change from baselines of % CD69, % CD25, % HLA-DR and % PD-1 on $CD4^+$ T_M in blood of rapamycin-treated RMs (n = 7) vs. vehicle controls (n = 7). The WRS test was used to determine the significance of differences in AUC between the two treatment groups.

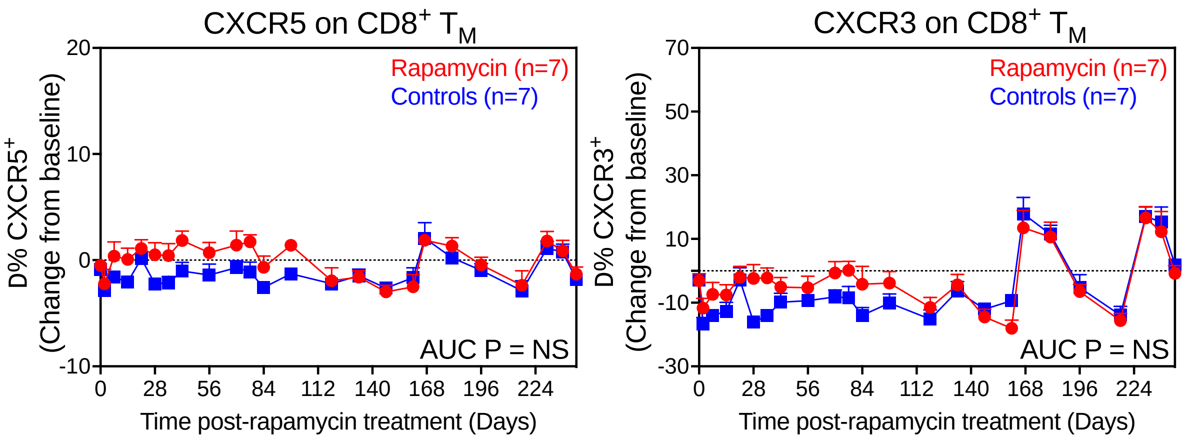

Supplementary Figure 10: Effect of rapamycin treatment on chemokine receptors on CD8^{+} T$_{M}$. Mean (+ SEM) change from baselines of % CXCR5 and % CXCR3 on CD8^{+} T$_{M}$ in blood of rapamycin-treated RMs (n = 7) vs. vehicle controls (n = 7). The WRS test was used to determine the significance of differences in AUC between the two treatment groups.

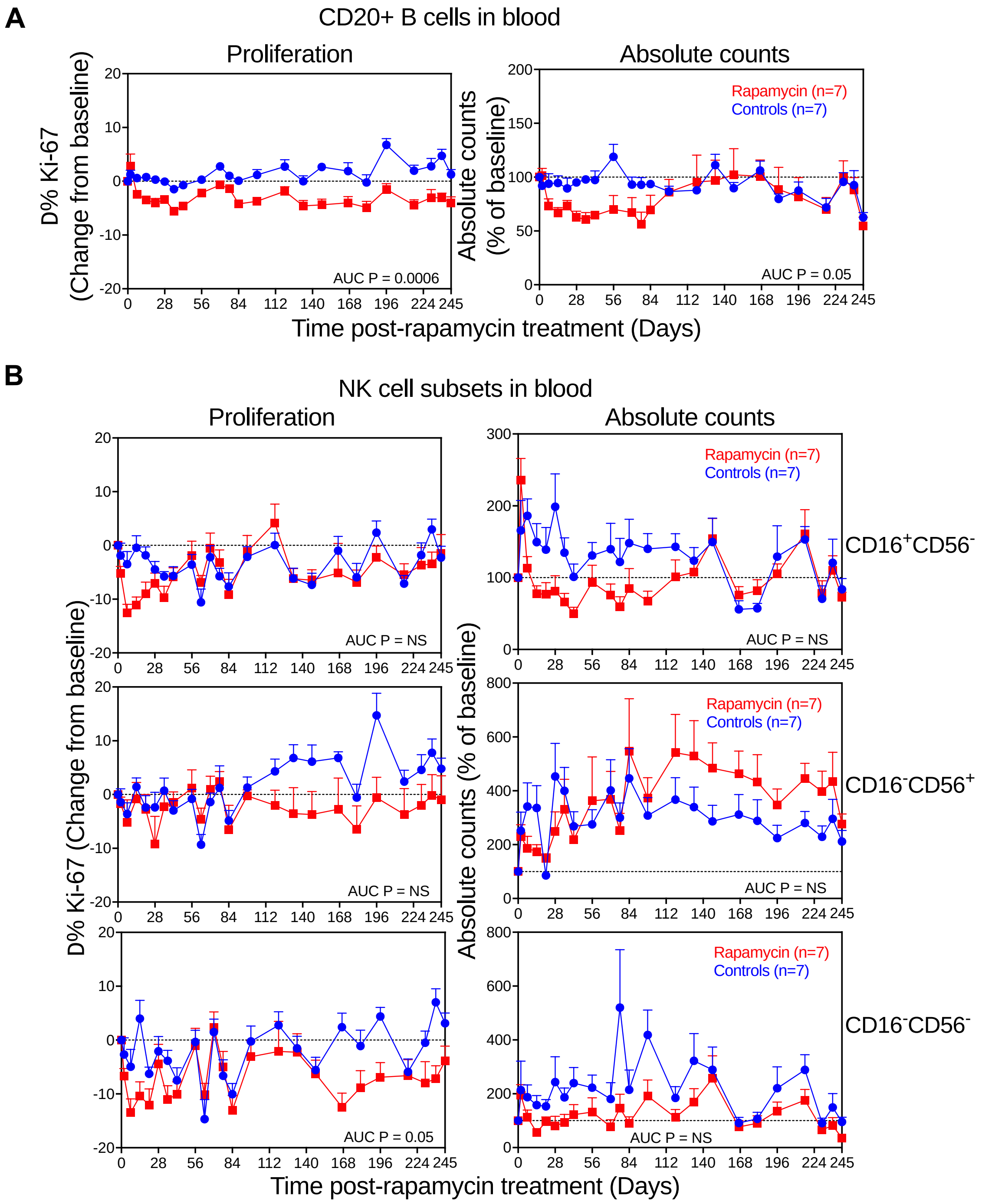

Supplementary Figure 11: Effect of rapamycin treatment on B cell and NK cell dynamics. (A) Change in the proliferative fraction (left panels) and absolute counts (right panels) of CD20$^+$ B cells in blood following rapamycin (n = 7) vs. vehicle control (n = 7) treatment. (B) Change in the proliferative fraction (left panels) and absolute counts (right panels) of NK cell subsets, including CD16$^+$CD56$^-$, CD16$^-$CD56$^+$ and CD16$^-$CD56$^-$ NK cells in blood following rapamycin (n = 7) vs. vehicle control (n = 7) treatment. Results are shown as mean (+ SEM) change from baseline of %Ki-67 (left panels) and % of baseline absolute counts (right panels). The WRS test was used to determine the significance of differences in AUC between the two treatment groups (p-values ≤ 0.05 are shown).

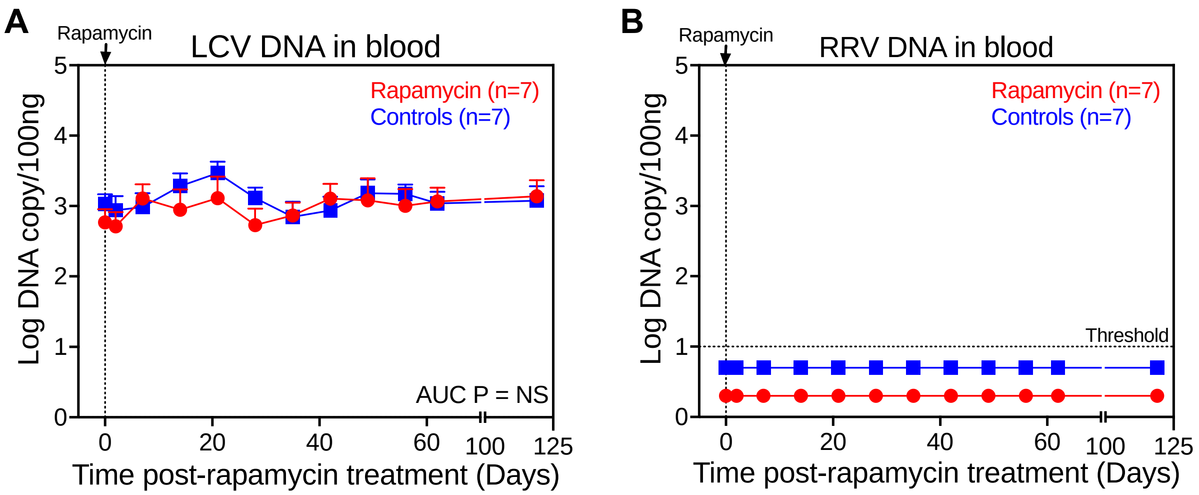

Supplementary Figure 12: Effect of rapamycin treatment on LCV and RRV dynamics. (**A**) Quantification of LCV DNA in the blood following rapamycin (n = 7) vs. vehicle control (n = 7) treatment. Results (mean + SEM) are shown as log LCV genome copies/100 ng DNA in each RM. (B) Quantification of RRV DNA in the blood following rapamycin (n = 7) vs. vehicle control (n = 7) treatment. Results (mean + SEM) are shown as log RRV genome copies/100 ng DNA in each RM. The WRS test was used to determine the significance of differences in AUC between the two treatment groups (p-values ≤ 0.05 are shown).

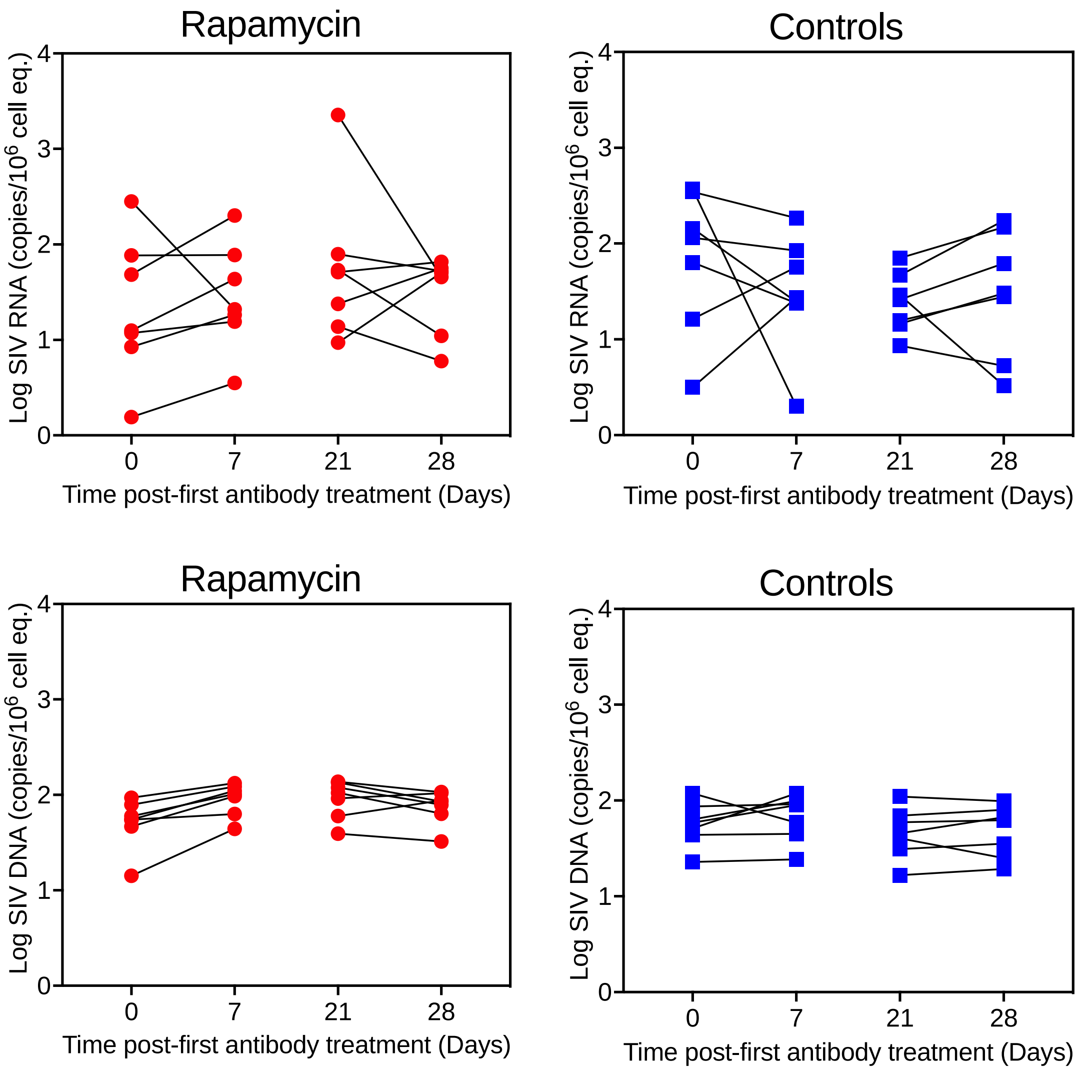

Supplementary Figure 13: Effect of anti-CD3LALA on cell-associated viral loads. Comparison of SIV RNA (top panels) and DNA (bottom panels) levels in PBMC (copies per 10^6 cell equivalents) between rapamycin (red, n = 7) or vehicle controls (blue, n = 7) at 0 and 7 days after each infusion of anti-CD3LALA or IgG control antibody, respectively. Each data point represents a single determination from an individual RM. The WRS test was used to determine the significance of differences between day 0 and 7 post-antibody infusion.

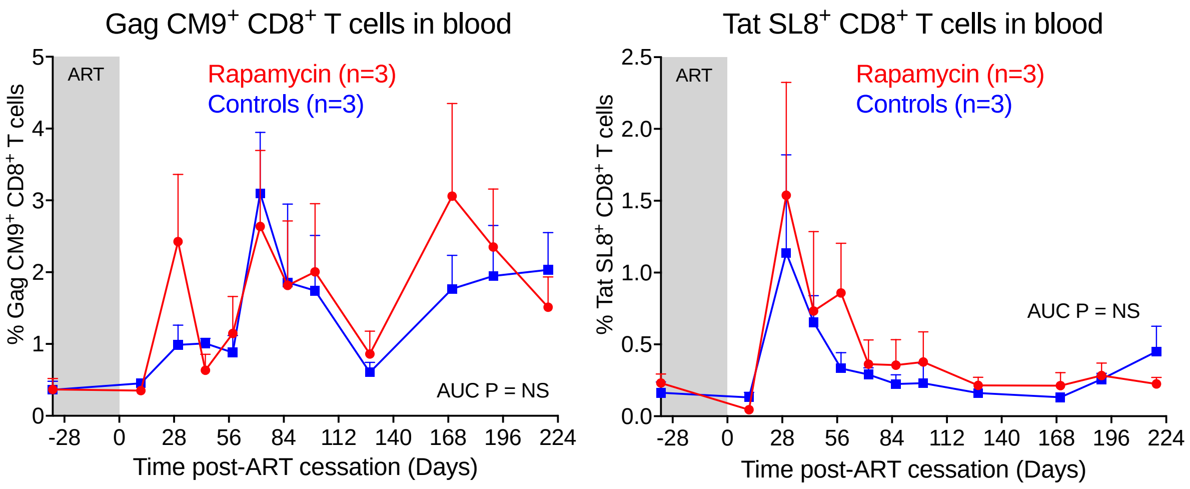

Supplementary Figure 14: Quantification of Gag-CM9^{+} (left panel) and Tat-SL8^{+} (right panel), CD8^{+} T cells in blood of 6 *Mamu* A*01 RM treated with rapamycin (red; n = 3) or vehicle control (blue; n = 3) after ART withdrawal. Results are shown as mean (+ SEM) % tetramer^{+} CD8^{+} T cells. The WRS test was used to determine the significance of differences in AUC between the two treatment groups (p-values ≤ 0.05 are shown).

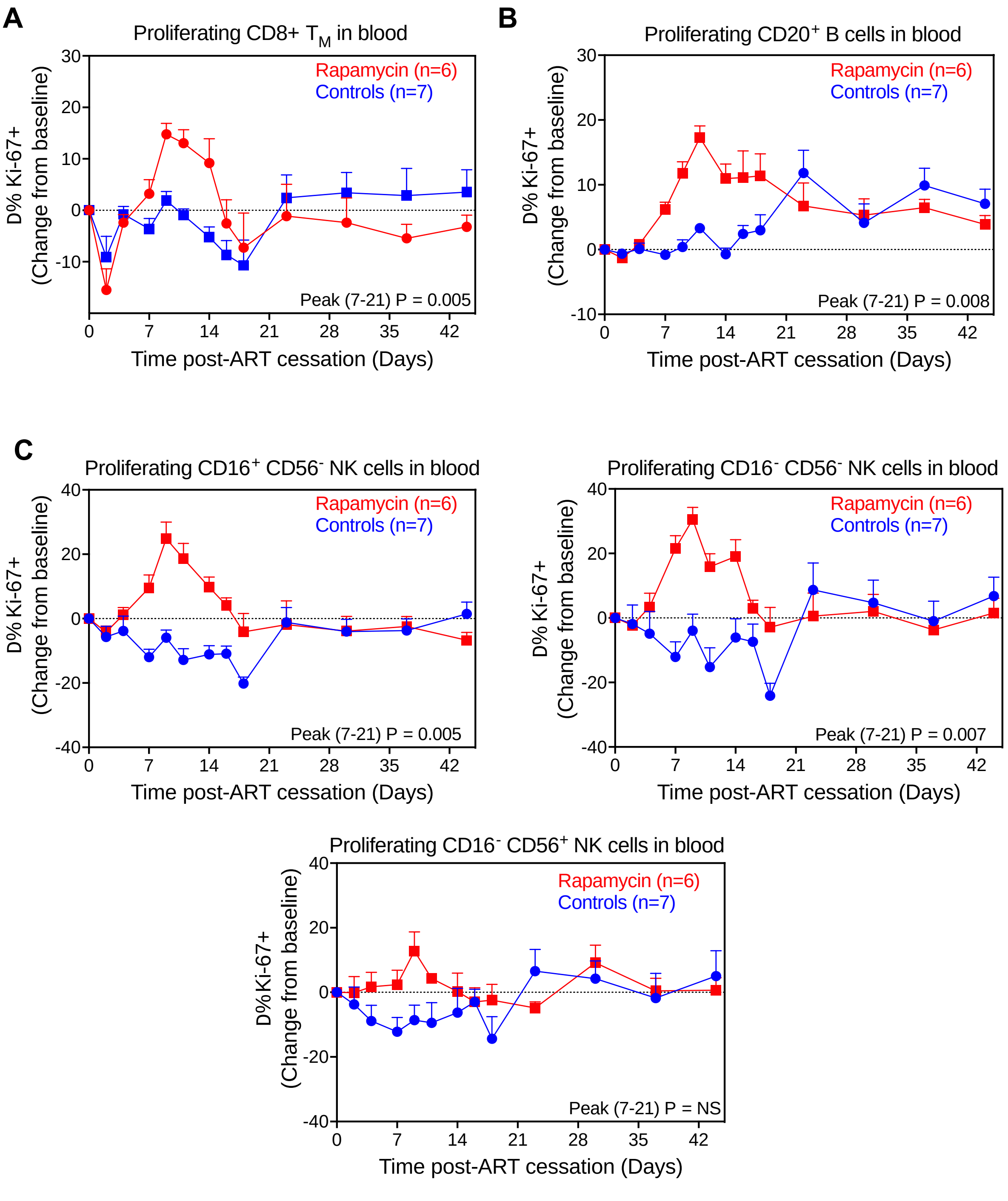

Supplementary Figure 15: Effect of rapamycin on CD8$^+$ T$_M$, B cell and NK cell proliferation after ART withdrawal. Change in the proliferative fraction of (**A**) CD8$^+$ T$_M$, (**B**) CD20$^+$ B cells and (**C**) NK cell subsets, including CD16$^+$CD56$^-$, CD16$^-$CD56$^+$ and CD16$^-$CD56$^-$ NK cells in blood of rapamycin treated RM (n = 6) vs. vehicle controls (n = 7) after ART withdrawal. Results are shown as mean (+ SEM) change from baseline of %Ki-67. The WRS test was used to determine the significance of differences in AUC between the two treatment groups (p-values $\leq$ 0.05 are shown). For these analyses n=13; one animal in the rapamycin group was lost from study just prior to ART withdrawal and was therefore not included in the final analysis.
